# The Role of Bone Morphogenetic Protein Signaling in Non-Alcoholic Fatty Liver Disease

**DOI:** 10.1038/s41598-020-66770-8

**Published:** 2020-06-19

**Authors:** Timothy E. Thayer, Christian L. Lino Cardenas, Trejeeve Martyn, Christopher J. Nicholson, Lisa Traeger, Florian Wunderer, Charles Slocum, Haakon Sigurslid, Hannah R. Shakartzi, Caitlin O’Rourke, Georgia Shelton, Mary D. Buswell, Hanna Barnes, Leif R. Neitzel, Clara D. Ledsky, Jason Pingcheng Li, Megan F. Burke, Eric Farber-Eger, Daniel S. Perrien, Ravindra Kumar, Kathleen E. Corey, Quinn S. Wells, Kenneth D. Bloch, Charles C. Hong, Donald B. Bloch, Rajeev Malhotra

**Affiliations:** 1 Cardiovascular Research Center and Cardiology Division of the Department of Medicine, Massachusetts General Hospital, Harvard Medical School, Boston, MA United States; 2 Anesthesia Center for Critical Care Research of the Department of Anesthesia, Critical Care, and Pain Medicine, Massachusetts General Hospital, Harvard Medical School, Boston, MA United States; 3 GI Unit, Massachusetts General Hospital, Harvard Medical School, Boston, MA United States; 4 Center for Immunology and Inflammatory Diseases and the Division of Rheumatology, Allergy, and Immunology of the Department of Medicine, Massachusetts General Hospital, Harvard Medical School, Boston, MA United States; 5 Acceleron Pharma, Inc., Cambridge, MA United States; 6 Department of Medicine, Vanderbilt University Medical Center, Nashville, TN United States; 7 Department of Medicine, University of Maryland School of Medicine, Baltimore, MD United States

**Keywords:** Mechanisms of disease, Non-alcoholic fatty liver disease, Experimental models of disease, Preclinical research

## Abstract

Non-alcoholic fatty liver disease (NAFLD) affects over 30% of adults in the United States. Bone morphogenetic protein (BMP) signaling is known to contribute to hepatic fibrosis, but the role of BMP signaling in the development of NAFLD is unclear. In this study, treatment with either of two BMP inhibitors reduced hepatic triglyceride content in diabetic (*db/db*) mice. BMP inhibitor-induced decrease in hepatic triglyceride levels was associated with decreased mRNA encoding Dgat2, an enzyme integral to triglyceride synthesis. Treatment of hepatoma cells with BMP2 induced DGAT2 expression and activity via intracellular SMAD signaling. In humans we identified a rare missense single nucleotide polymorphism in the BMP type 1 receptor *ALK6* (rs34970181;R371Q) associated with a 2.1-fold increase in the prevalence of NAFLD. *In vitro* analyses revealed R371Q:ALK6 is a previously unknown constitutively active receptor. These data show that BMP signaling is an important determinant of NAFLD in a murine model and is associated with NAFLD in humans.

## Introduction

The prevalence of non-alcoholic fatty liver disease (NAFLD) in adults exceeds 30% in Western countries and NAFLD portends increased risk of cardiovascular disease^1–3^. The prevalence of NAFLD is particularly high among individuals with diabetes mellitus, exceeding 70% in some populations^4,5^. NAFLD encompasses a spectrum of disorders ranging from simple hepatic steatosis without inflammation to steatosis with inflammation (non-alcoholic steatohepatitis) to hepatic fibrosis and cirrhosis^6,7^. Despite the enormous scale of morbidity and mortality associated with NAFLD, treatments are limited to risk factor modification including lifestyle intervention and management of co-morbid metabolic disease. Currently, there are no FDA-approved pharmacotherapies for NAFLD.

The pathophysiology of NAFLD involves the accumulation of neutral lipids, predominantly triglycerides, in the liver^8^. Diacylglycerol O-acyltransferase 2 (DGAT2) catalyzes the conversion of diacylglycerol (DAG) to triglyceride^9^. DAG is the precursor for many end products and DGAT2 is responsible for committing hepatic DAG to triglyceride^10,11^. Altering DGAT2 expression or activity *in vivo* via pharmacologic inhibition or genetic knockdown, knockout, or overexpression leads to alterations in hepatic triglyceride content^12–18^. Thus, DGAT2 expression is thought to be central to the hepatic accumulation of triglyceride in NAFLD.

Bone morphogenetic protein (BMP) signaling is involved in the pathophysiology of hepatic fibrosis, however the role of BMP signaling in the pathophysiology of simple steatosis has not been well elucidated^19–21^. BMP ligands are members of the transforming growth factor-beta superfamily and act as ubiquitous paracrine messengers^19,21^. Canonical BMP signaling occurs through heterotetrameric type I (ALK2, ALK3, and ALK6) and type II receptors that activate intracellular SMAD 1, 5, & 8 (SMAD 1/5/8) transcription factors. SMAD 1/5/8 regulate the expression of many downstream genes, including inhibitor of DNA binding 1 (ID1)^22,23^.

In this study, we treated diabetic *db/db* mice with BMP inhibitors to investigate the role of BMP signaling in the development of simple hepatic steatosis. The *db/db* mouse spontaneously develops hepatic steatosis by four weeks of age and is a well-established model of NAFLD^24–26^. We report three novel findings that highlight the importance of BMP signaling in the development of NAFLD: treating *db/db* mice with BMP inhibitors significantly reduces hepatic steatosis; BMP signaling regulates hepatic DGAT2 expression and activity; and a rare single nucleotide polymorphism (SNP) in a BMP type I receptor results in constitutive activation and is associated with NAFLD in humans. Together, these results provide *in vivo*, *in vitro*, and human genetic evidence for the role of BMP signaling in NAFLD and suggest a potential role for drugs targeting the BMP signaling pathway in the treatment of NAFLD.

## Results

### Pharmacologic inhibition of BMP signaling reduces hepatic steatosis

**Figure 1:**
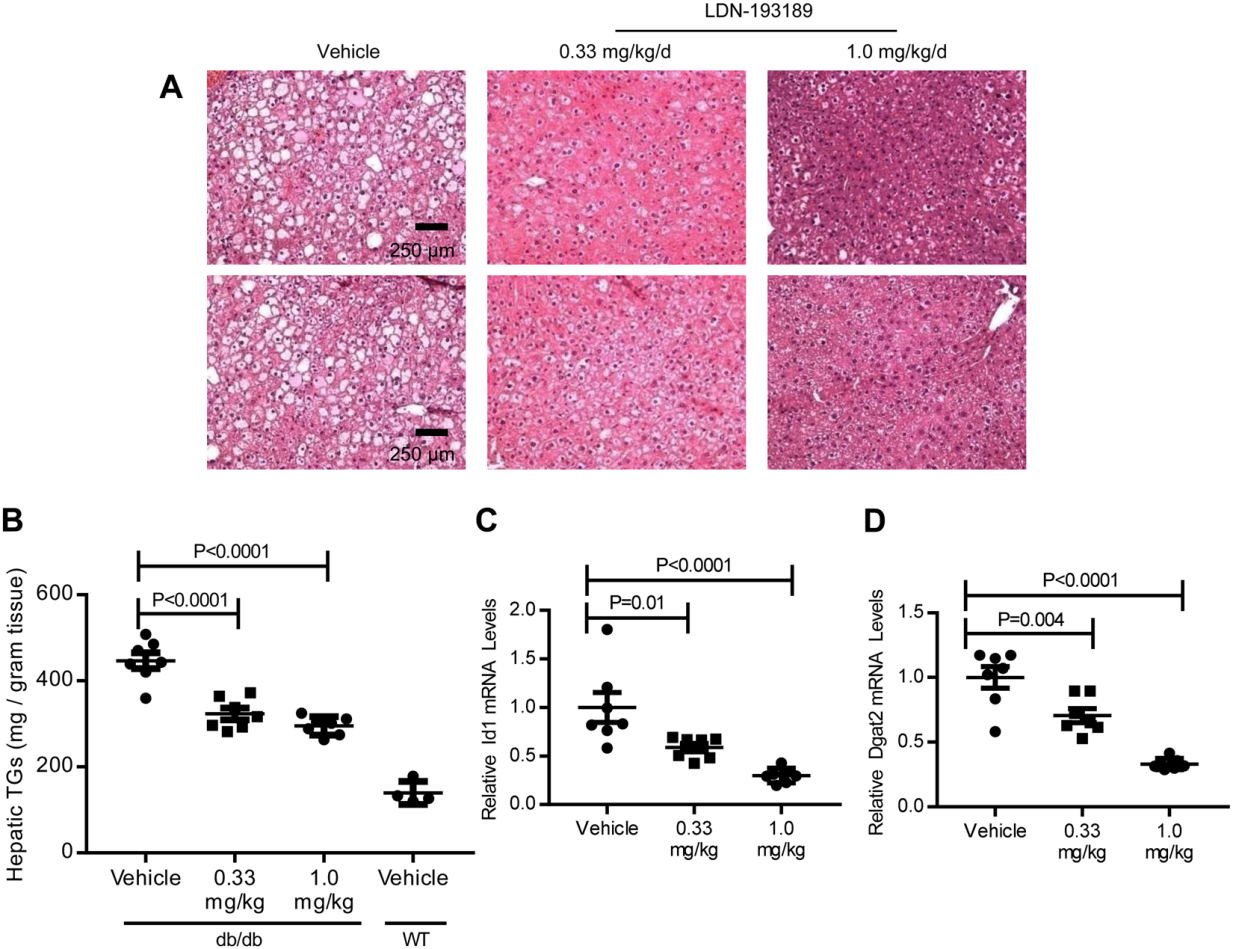
Pharmacologic inhibition of BMP signaling with LDN-193189 reduces hepatic steatosis. Representative photomicrographs of liver sections from leptin receptor-deficient (*db/db*) mice treated with vehicle or the BMP inhibitor LDN-193189 for 14 days beginning at 4 weeks of age. Sections were stained with hematoxylin and eosin (**A**). LDN-193189 treatment reduced hepatic tissue triglyceride (TG) toward wild-type (WT) levels (**B**). Treatment of *db/db* mice with LDN-193189 reduced hepatic BMP signaling as measured by inhibitor of DNA binding 1 (Id1) mRNA levels and was associated with reduced diacylglycerol O-acyltransferase 2 (Dgat2) mRNA levels (**C**,**D** respectively). There were seven replicates per treatment group. Comparisons were performed using 1-way ANOVA with Sidak’s multiple comparison testing. Scale bars represent 250 µm and apply to all six photomicrographs.

To investigate the role of BMP signal transduction in a mouse model of hepatic steatosis, obese *db/db* mice were treated with LDN-193189, an inhibitor of BMP type I receptor signal transduction^27^. Mice received low-dose (0.33 mg/kg/day) or high-dose (1.0 mg/kg/day) LDN-193189, or vehicle alone, for 14 days. Compared to vehicle-treated *db/db* mice, *db/db* mice treated with either low-dose or high-dose LDN-193189 had decreased hepatic steatosis, as determined by histologic examination of the liver (Fig. 1A) and by measurement of hepatic triglyceride levels (Fig. 1B). Relative to vehicle-treated *db/db* mice, treatment of *db/db* mice with LDN-193189 decreased hepatic triglyceride levels by 28% (low-dose LDN-193189: 0.33 mg/kg, P < 0.0001) and 34% (high-dose LDN-193189: 1.0 mg/kg, P < 0.0001). The reduction in hepatic triglyceride levels correlated with an LDN-193189 dose-dependent reduction in hepatic *Id1* mRNA levels, which confirmed inhibition of BMP signaling (Fig. 1C). Protein blots of phosphorylated SMAD also demonstrated that LDN-193189 treatment successfully inhibited BMP signaling (Supplemental Fig. 1). Compared to control animals, treatment with LDN-193189 did not alter the body weight of *db/db* mice (Supplemental Fig. 2A). Treatment of *db/db* mice with LDN-193189 resulted in a dose-dependent reduction in hepatic *Dgat2* mRNA levels (Fig. 1D). Hepatic mRNA levels of other proteins involved in hepatic triglyceride synthesis or lipid transport were not altered by treatment with both low and high dose LDN-193189 (Supplemental Fig. 3)^10,28,29^. Thus, treatment of *db/db* mice with a small molecule inhibitor of BMP signaling reduced hepatic steatosis and *Dgat2* mRNA in a dose-dependent fashion.

### LDN-193189-mediated inhibition of BMP signaling reduced plasma markers of hepatic injury without affecting total cholesterol levels or glucose tolerance

Alanine aminotransferase and alkaline phosphatase were lower in *db/db* mice treated with LDN-193189 in a dose-dependent fashion, indicating reduced hepatic injury (Table 1). Similarly, immunofluorescence staining of liver sections from vehicle- and LDN-193189-treated mice showed a stepwise reduction in markers of inflammation, interleukin 1-beta and matrix metalloproteinase-9 (Supplemental Fig. 4). The levels of mRNA encoding other inflammatory and fibrotic proteins were not affected by LDN-193189 treatment (Supplemental Fig. 5). Treatment of *db/db* mice with LDN-193189 also reduced plasma triglyceride levels by up to 50% (Table 1). Total cholesterol levels did not differ between LDN-193189 and vehicle-treated mice and there was no difference in glucose tolerance between the two groups (Supplemental Fig. 6). Thus, BMP inhibition reduced plasma markers of hepatic steatosis independent of plasma cholesterol levels and glucose tolerance.

**Table 1:** Plasma markers of hepatic injury, cholesterol, and triglyceride levels in *db/db* mice treated with either vehicle or the BMP signaling inhibitor LDN-193189.

Plasma Test	Vehicle (n = 7)	LDN-193189 @ 0.33 mg/kg/d (n = 7)	LDN-193189 @ 1.0 mg/kg/d (n = 7)	ANOVA P value
Alanine aminotransferase (U/L)	88 ± 6	66 ± 8*	43 ± 3*	0.0003
Alkaline phosphatase (U/L)	107 ± 5	94 ± 6	77 ± 5*	0.003
Total cholesterol (mg/dL)	95 ± 4	96 ± 7	110 ± 6	0.15
Triglyceride (mg/dL)	297 ± 49	190 ± 18*	148 ± 21*	0.01

Mean ± SEM; Trend P value from a 1-way ANOVA is indicated.

*P < 0.05 compared to vehicle

### Treatment of *db/db* mice with ALK3-Fc, an alternative inhibitor of BMP signaling, also reduced hepatic steatosis

**Figure 2:**
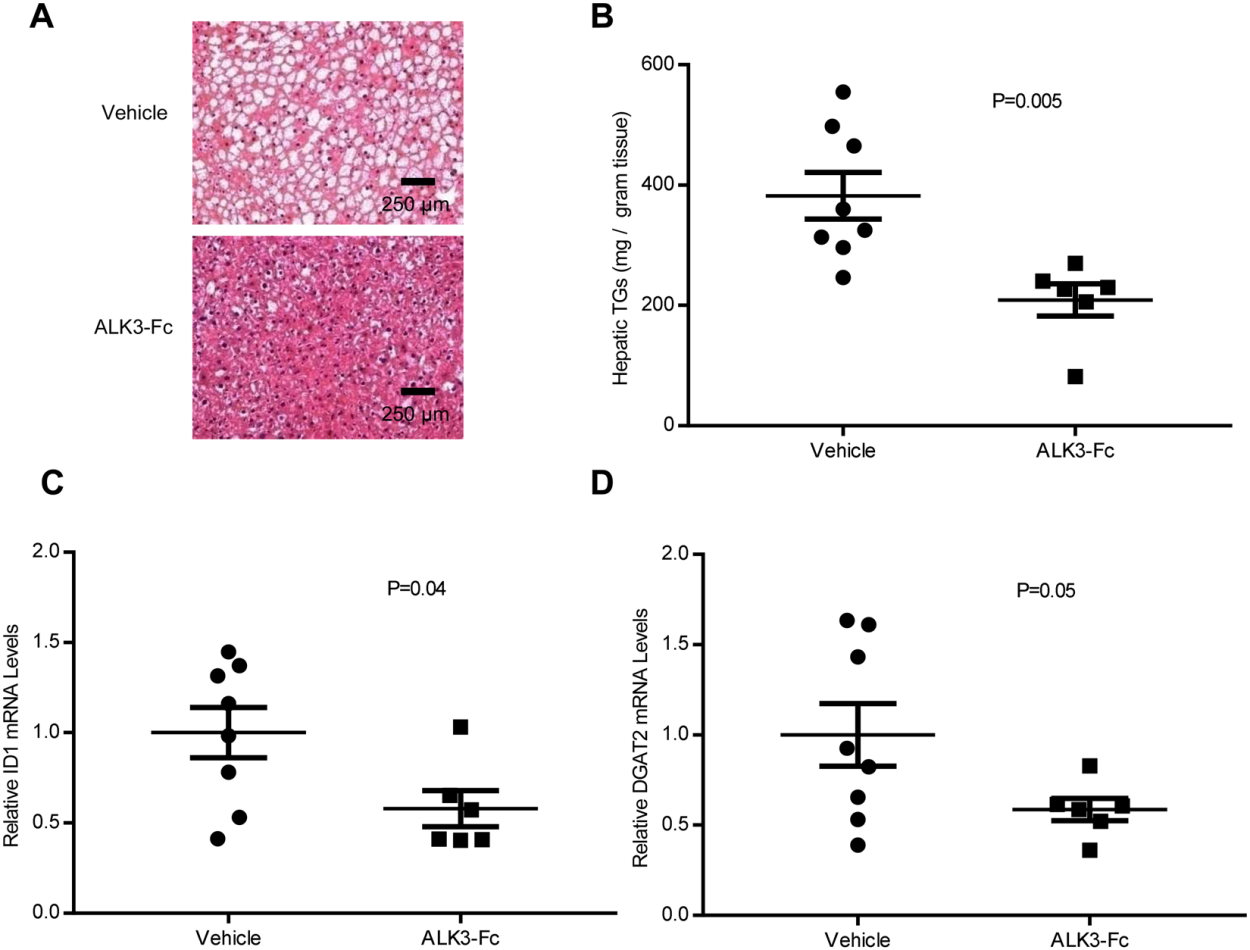
Pharmacologic inhibition of BMP signaling with ALK3-Fc reduces hepatic steatosis. Representative photomicrographs of liver sections from *db/db* mice treated with vehicle or the BMP inhibitor ALK3-Fc for 21 days beginning at 4 weeks of age. Sections were stained with hematoxylin and eosin (**A**). ALK3-Fc treatment reduced hepatic triglyceride levels (**B**). Treatment of *db/db* mice with ALK3-Fc reduced hepatic BMP signaling as measured by Id1 (inhibitor of DNA binding 1) mRNA levels and was associated with reduced Dgat2 (diacylglycerol O-acyltransferase 2) mRNA levels (**C**,**D** respectively). There were six replicates in the ALK3-Fc treatment group and eight replicates in the vehicle treatment group. Comparisons were performed using the two-tailed Student’s t test. Scale bars represent 250 µm.

ALK3-Fc binds to BMP ligands and sequesters them from BMP receptors, thereby down-regulating BMP signaling through a mechanism that is distinct from that of LDN-193189. Similar to LDN-193189, ALK3-Fc decreased hepatic steatosis in *db/db* mice (Fig. 2A). Treatment with ALK3-Fc did not affect the body weight of *db/db* mice (Supplemental Fig. 2B). Compared to vehicle-treated *db/db* mice, ALK3-Fc decreased hepatic triglyceride levels by 45% (P = 0.005, Fig. 2B). Inhibition of BMP signaling by ALK3-Fc also reduced hepatic *Id1* and *Dgat2* mRNA levels by 42% (Fig. 2C, P = 0.04) and 41% (Fig. 2D, P = 0.05), respectively. BMP inhibition with ALK3-Fc was confirmed by reduced levels of phosphorylated SMAD (pSMAD)1/5/8 in the livers of ALK3-Fc-treated mice (Supplemental Fig. 7). Taken together, the results of studies using two inhibitors of BMP signaling (LDN-193189 and ALK3-Fc) with different mechanisms of action, demonstrate the important role of BMP signaling in the development of hepatic steatosis in *db/db* mice.

### In an *in vitro* model of hepatic steatosis, lipid accumulation and DGAT2 expression is dependent on BMP signaling

**Figure 3:**
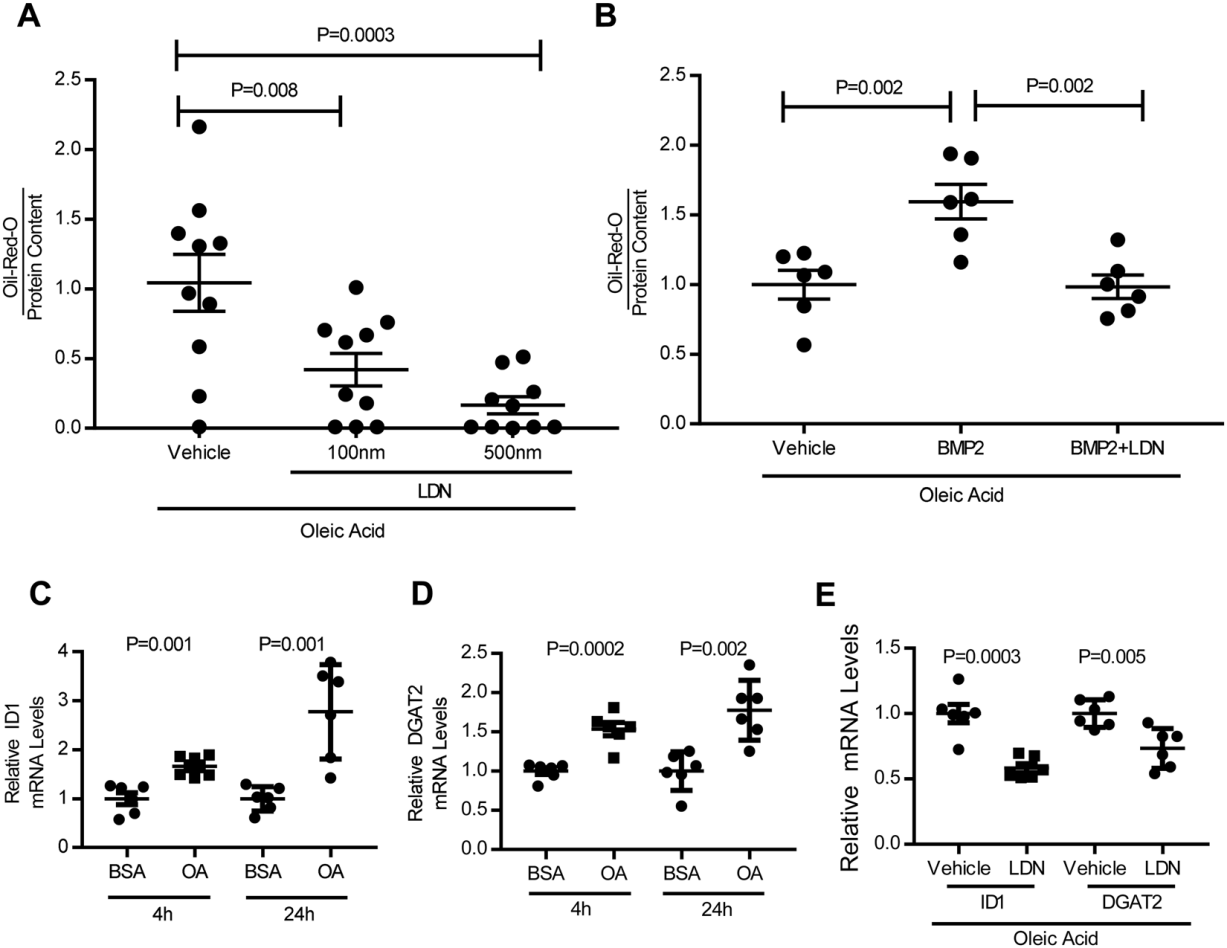
*In vitro* hepatocyte lipid accumulation is induced by BMP signaling and is prevented by BMP inhibition. Treatment of HepG2 cells (grown in media containing 1.2 mM oleic acid (OA)) for 24 h with increasing concentrations of LDN-193189 had reduced intracellular lipid content compared to vehicle-treated cells as assessed by measurement of Oil-Red-O staining normalized to total protein content (**A**, 10 replicates per group). Similarly, HepG2 cells (grown in media containing OA) treated with BMP2 (20 ng/ml) had increased intracellular lipid content and this induction was reversed by co-treatment with LDN-193189 (**B**, six replicates per group). Treatment of HepG2 cells with OA for 4 h or 24 h, compared to control cells treated for the same durations with bovine serum albumin (BSA) induced mRNA expression of *ID1* (inhibitor of DNA binding 1) in HepG2 cells (**C**, six replicates per group). Similarly, incubation of HepG2 cells with OA also induced *DGAT2* (diacylglycerol O-acyltransferase 2) expression at 4 h and 24 h (**D**, six replicates per group). *ID1* and *DGAT2* expression were both inhibited by treatment with LDN-193189 in HepG2 cells (**E**, six replicates per group). Comparisons were performed using the 1-way ANOVA with Sidak’s multiple comparison testing for (**A**,**B**) and two-tailed Student’s t test for (**C**–**E**).

An established *in vitro* model of steatosis was used to further investigate the role of BMP signaling in hepatocyte lipid accumulation^30^. Human hepatocellular carcinoma (HepG2) cells were incubated with oleic acid to induce lipid accumulation (predominantly triglycerides) and Oil-Red-O stain was used to quantify the level of intracellular lipids. LDN-193189 treatment resulted in a dose-dependent reduction in intracellular lipid levels (Fig. 3A). BMP2 increased lipid levels in HepG2 cells and this effect was reversed by the addition of LDN-193189 (Fig. 3B). Treatment of HepG2 cells with oleic acid for either 4 or 24 hours induced BMP ligand, *ID1*, and *DGAT2* gene expression in HepG2 cells (Supplemental Fig. 8, Fig. 3C,D, respectively). LDN-193189 inhibited oleic acid-induced *DGAT2* expression in HepG2 cells (Fig. 3E). Thus, oleic acid-induced lipid accumulation and DGAT2 expression are modified by BMP signaling in this *in vitro* model of hepatic steatosis.

### *In vitro* stimulation of hepatoma cell lines with exogenous BMP ligand induces DGAT2 expression and activity

**Figure 4:**
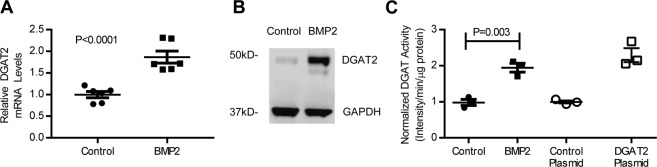
BMP2 increases DGAT2 expression and activity. Treatment of HepG2 cells with BMP2 resulted in increased expression of *DGAT2* (diacylglycerol O-acyltransferase 2) mRNA (**A**, six replicates per group) as well as increased DGAT2 protein levels at 24hrs (**B**). In parallel experiments, DGAT activity was assessed by quantification of fluorescently-labeled triglyceride production in HepG2 cells treated with BMP2 or control at 24hrs with DGAT2 overexpression and control plasmid as additional controls (**C**, three replicates per group). Comparisons were made using the two-tailed Student’s t test.

Because pharmacologic inhibition of BMP signaling reduced *Dgat2* expression in *db/db* mice and in HepG2 cells treated with oleic acid, we sought to determine whether increased BMP signaling induces *DGAT2* expression. HepG2 cells were treated with either BMP2 or vehicle alone for 24 hours. Compared to vehicle-treated HepG2 cells, BMP2 induced a 1.9-fold increase in *DGAT2* mRNA (P < 0.0001, Fig. 4A). The BMP2-induced increase in *DGAT2* mRNA was associated with an increase in DGAT2 protein expression (Fig. 4B). Stimulation of HepG2 cells with other BMP ligands also induced *DGAT2* expression (Supplemental Fig. 9). RNA expression of other important mediators of lipid metabolism, including DGAT1, FAS, and CPT1 were not affected by BMP stimulation (Supplemental Fig. 10). The results show that treatment of HepG2 cells with BMP2 leads to increased expression of DGAT2.

To test the hypothesis that increased BMP signaling promotes *DGAT2* expression in hepatoma cell lines other than HepG2, we investigated whether BMP signaling increased *DGAT2* expression in HuH7 cells (a human hepatoma cell line), primary human hepatocytes, and AML12 mouse hepatocytes. Consistent with the results observed in HepG2 cells, treatment of HuH7 cells, AML12 cells, and primary human hepatocytes with BMP2 for 24 hours increased *DGAT2* mRNA levels (Supplemental Fig. 11).

To investigate the effect of BMP signaling on DGAT2 activity, HepG2 cells were stimulated with either BMP2 or vehicle for 24 hours. Membrane protein fractions were isolated and incubated with fluorescently-labeled palmitoyl-CoA. Free fatty acids and triglycerides were then separated using TLC plates and the triglyceride fraction was quantified as a measurement of DGAT2 activity. Compared to membranes prepared from control cells, membranes from BMP2-stimulated cells had ~2-fold increased DGAT2 activity (P = 0.003, Fig. 4C).

### BMP-induced DGAT2 expression is SMAD dependent

**Figure 5:**
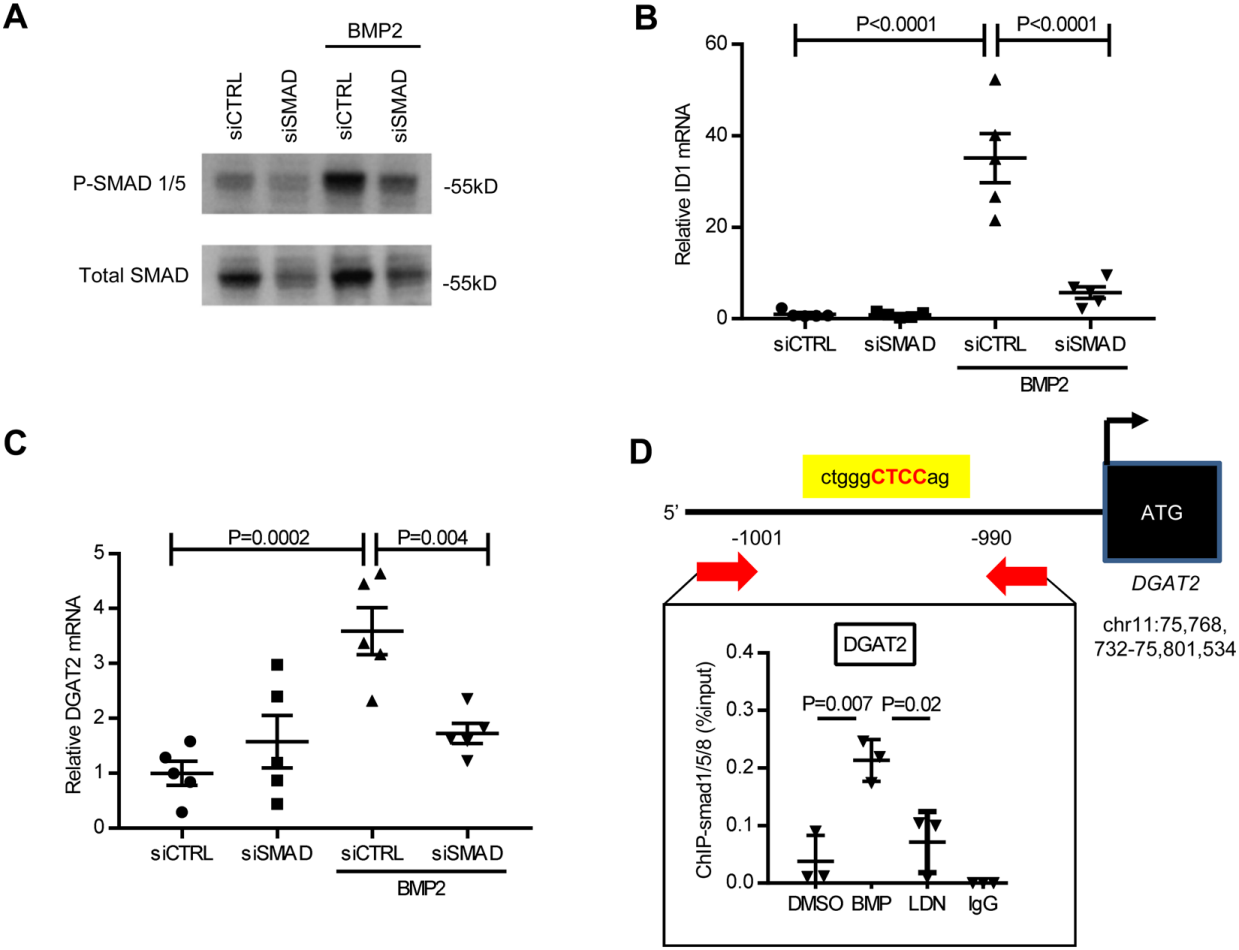
BMP-induced *DGAT2* (diacylglycerol O-acyltransferase 2) expression is mediated through SMAD signaling. HepG2 cells treated with siRNA directed against SMAD 1/5 had reduced SMAD 1/5 protein levels (**A**) and decreased canonical BMP signaling as evidenced by decreased mRNA expression of *ID1* (inhibitor of DNA binding 1) when stimulated with the BMP ligand BMP2 (**B**). BMP2-induced *DGAT2* expression is SMAD1/5 dependent (**C**). Using chromatin immunoprecipitation combined with qPCR (ChIP-qPCR), BMP2 stimulation resulted in increased binding of SMAD 1/5 to a putative SMAD promoter region (1000 base pairs upstream of the DGAT2 start site) at 10-fold higher levels compared to control levels (**D**). Experiments had five replicates per group for panels (**B,C**) and three replicates per group for panel (**D**). SMAD western blots were run on 2 separate gels given similar protein weights. Comparisons were performed using 1-way ANOVA with Sidak’s multiple comparison testing.

BMP signaling can occur through SMAD-dependent and SMAD-independent intracellular messengers. To investigate whether BMP-induced DGAT2 expression is dependent on SMAD proteins, we used small inhibitory RNAs specific for the mRNAs that encode the redundant intracellular proteins SMAD1 and SMAD5 (siSMAD) to reduce SMAD protein levels. HepG2 cells were treated with either siSMAD or control siRNA (siCTRL) in the presence or absence of BMP2. In the absence of BMP2, treatment with siSMAD reduced SMAD protein levels in HepG2 cells by 40% (Fig. 5A). SMAD knockdown reduced BMP2-induced *ID1* mRNA levels (Fig. 5B), confirming successful inhibition of the canonical BMP signaling pathway. In control siRNA-treated HepG2 cells, BMP2 increased *DGAT2* mRNA levels by 3.6-fold. Knockdown of SMAD1 and SMAD5 resulted in a 70% reduction in the BMP2-induced increase in *DGAT2* mRNA (Fig. 5C).

We hypothesized that the BMP-induced increase in *DGAT2* mRNA levels occurs via a SMAD promoter-binding region upstream of the DGAT2 coding sequence. A consensus SMAD binding sequence was located 1000 base pairs upstream of the DGAT2 start site. Using chromatin immunoprecipitation combined with qPCR (ChIP-qPCR), BMP2 treatment of HepG2 cells produced a 10-fold increase in binding of SMAD1/5 to the putative promoter region (Fig. 5D). These results show that BMP2-induced *DGAT2* expression is SMAD-dependent with increased binding of SMAD1/5 to the DGAT2 promoter.

### The ALK6 variant rs34970181 is associated with non-alcoholic and non-infectious liver disease

A screening candidate gene analysis was performed to determine whether missense variants in BMP type 1 receptors are associated with the prevalence of non-alcoholic and non-infectious liver disease in humans. Using BioVU (n~20,000 participants), the Vanderbilt University Medical Center DNA biorepository linked to de-identified electronic health records, six missense SNPs were identified in the BMP type 1 receptors *ALK2*, *ALK3*, and *ALK6* with minor allele frequency (MAF) of >0.05% (Supplemental Table 2)^31–33^. In an initial screening analysis, these six SNPs were tested individually by logistic regression analysis for association with a pre-existing diagnosis in the electronic health record (PheCode 571.5). PheCode 571.5 is an aggregate of ICD9 codes for non-alcoholic and non-infectious chronic liver diseases, of which NAFLD is the most prevalent in the general population. After correction for false discovery rate, one low frequency variant in ALK6, rs34970181 (minor allele A with MAF 0.14%), was significantly associated with the presence of the PheCode 571.5 (OR = 3.24, 95% CI 2.2 to 4.4, P = 0.007). The minor allele variant of rs34970181 results in an amino acid substitution (R371Q) in the kinase domain of the ALK6 receptor^34^. This candidate gene study revealed that the minor allele of rs34970181 is associated with increased prevalence of non-alcoholic and non-infectious chronic liver disease.

### The ALK6 variant rs34970181 is associated with NAFLD

Because the PheCode 571.5 is not specific for NAFLD, the relationship between NAFLD and rs34970181 was directly assessed via a retrospective cohort analysis. Carriers of the rs34970181 minor allele were matched by age, sex, race, and BMI with non-carriers in a ratio of 1:2 as shown in Table 2. The presence of NAFLD was determined by review of the electronic health record by a physician who was blinded to rs34970181 status. NAFLD was diagnosed by imaging and/or biopsy-proven fatty liver and the absence of excess alcohol consumption or alternative diagnoses associated with hepatic steatosis. The rs34970181 minor allele was associated with NAFLD at more than twice the prevalence as matched controls (21.4% versus 10%, P = 0.03, Table 2). There was no evidence of ascertainment bias because liver imaging results and biopsy rates were similar in carriers and non-carriers: liver imaging was available for 60% of minor allele carriers and 58% of controls (P = 0.88); one case of NAFLD in rs34970181_A carriers and three cases in non-carriers were diagnosed by biopsy. Patients were excluded from analysis if other causes of fatty liver were present: five were excluded due to excessive alcohol consumption and one due to the presence of hepatitis C infection (2 minor allele carriers and 4 non-carriers). The presence of diabetes as well as the levels of serum total cholesterol, LDL, HDL, alanine aminotransferase, and aspartate aminotransferase levels were similar between the two groups. There was a trend toward increased serum triglycerides in rs34970181 minor allele carriers, although this did not reach statistical significance (P = 0.07). Taken together, the results show that carriers of rs34970181_A, a SNP in the BMP type 1 receptor gene *ALK6*, have increased odds of having NAFLD despite having similar BMI, diabetes prevalence, and lipid levels.

**Table 2:** The human ALK6 variant rs34970181_A was associated with NAFLD in a retrospective cohort study. Data from human carriers of rs34970181_A and matched non-carriers are presented. Matched characteristics are shown in the bottom section including age, BMI, sex, and race. Unmatched characteristics are shown in the top section. NAFLD status was determined by blinded physician chart review. Diabetes status was determined by ICD coding of the disease. Reported lab values are means of lifetime median values. Comparisons were performed using Fisher’s exact test for categorical data and Student’s t test for continuous variables.

Phenotype	rs34970181_A (n = 70)	Non-carrier (n = 140)	P-value
NAFLD	21.4%	10.0%	0.03
Diabetes	13.8%	10.1%	0.49
Total Cholesterol (mg/dL)	182 ± 6	181 ± 4	0.93
LDL (mg/dL)	103 ± 5	103 ± 3	0.99
HDL (mg/dL)	51 ± 3	53 ± 2	0.51
Triglycerides (mg/dL)	146 ± 5	129 ± 6	0.07
ALT (U/L)	25 ± 2	28 ± 4	0.53
AST (U/L)	27 ± 2	27 ± 1	0.72
**Age (years)**	**66 ± 0.3**	**66 ± 0.1**	**0.95**
**BMI (kg/m**^**2**^)	**28.2 ± 0.5**	**28.4 ± 0.7**	**0.89**
**Female**	**38.6%**	**37.5%**	**0.76**
**White**	**93.1%**	**93.1%**	**1**

Mean ± SEM for continuous variables

Abbreviations: Low-density lipoprotein (LDL); High-density lipoprotein (HDL); alanine aminotransferase (ALT); aspartate aminotransferase (AST).

### The R371Q ALK6 variant is constitutively active

**Figure 6:**
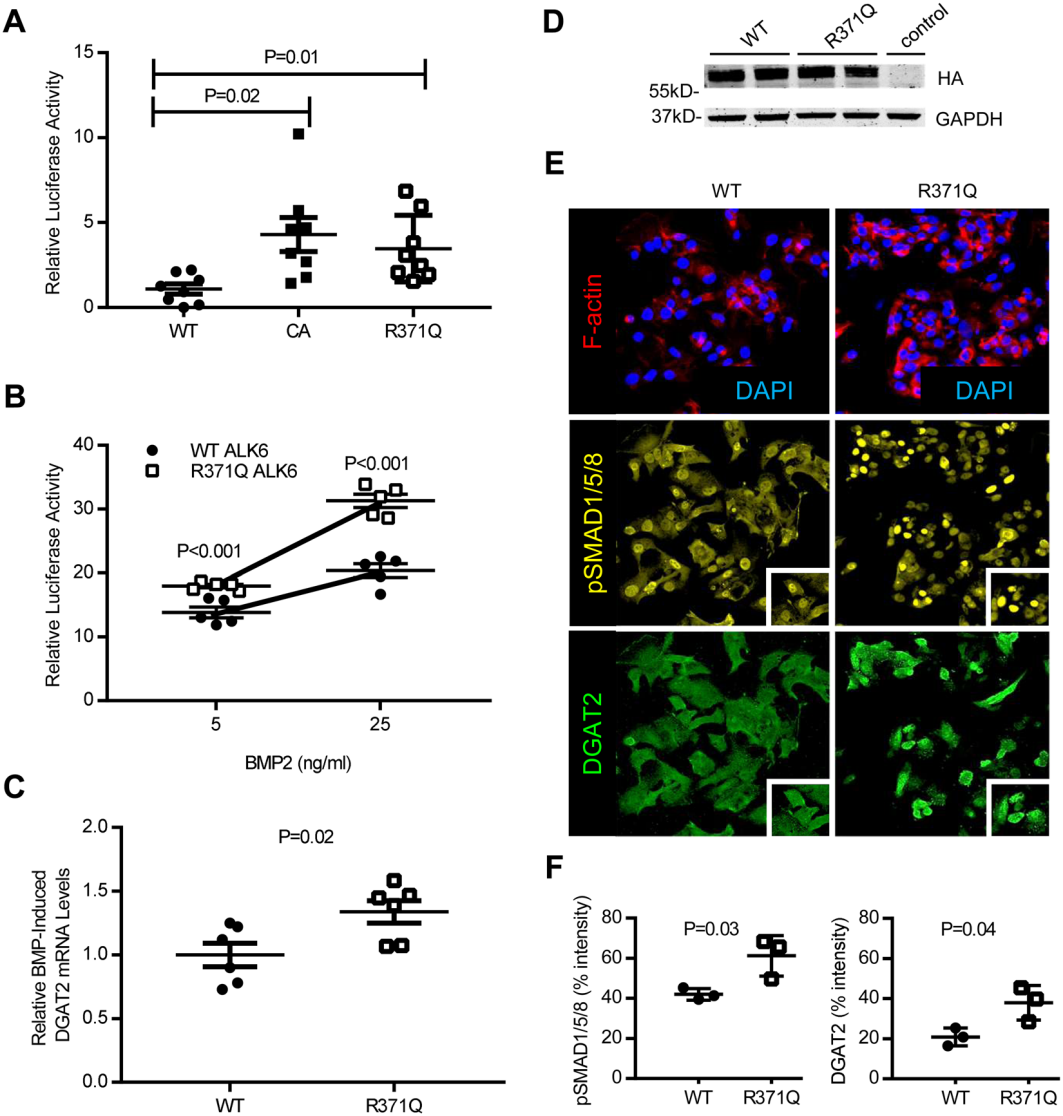
The ALK6 (Activin Receptor-Like Kinase 6) mutant R371Q is a constitutively active BMP type 1 receptor that is hyper-responsive to BMP ligands. C2C12BRA cells were stably transfected encoding a luciferase downstream of the BMP response element promoter (BRE-LUC), as well as either wild-type ALK6 (WT), a constitutively active ALK6 (CA), or R371Q ALK6. The ALK6 mutant R371Q (which results from the rs34970181_A variant) had increased baseline BMP signaling activity compared to WT ALK6 (**A**, eight replicates per group). Compared to WT ALK6, the R371Q ALK6 variant had increased BMP signaling in response to BMP2 (**B**, five replicates per group). Compared to WT ALK6, expression of the R371Q ALK6 variant in HepG2 cells resulted in increased BMP-induced *DGAT2* (diacylglycerol O-acyltransferase 2) mRNA levels (**C**, six replicates per group). Equal levels of expression of WT and R371Q ALK6 was confirmed by Western blot using antibodies detecting the HA epitope tag (**D**). GAPDH was used as a loading control. Immunofluorescence staining of HepG2 cells for pSMAD1/5/8 and DGAT2 transfected with wild-type ALK6 or R371Q ALK6 (**E**) and quantified using quantitative microscopy (**F**, % intensity represents signal of fluorophore vs background). The comparisons for (**A**) were performed using a 1-way ANOVA with Sidak’s multiple comparison testing and, for (**B**,**C**,**F**), two-tailed Student’s t test was used.

To investigate the functional significance of the missense variant rs34970181 *in vitro*, a plasmid encoding the ALK6 R371Q variant was prepared from a plasmid encoding wild-type ALK6 using site-directed mutagenesis. The plasmid encoding ALK6 R371Q was transfected into C2C12BRA mouse myoblast cells that stably expressed a luciferase reporter gene driven by a BMP response element (BRE). ALK6 R371Q transfection resulted in a 3.5-fold increase in relative luciferase activity compared to cells transfected with the wild-type ALK6 receptor (Fig. 6A). Expression of the ALK6 R371Q variant resulted in approximately the same increase in relative BRE-luciferase activity as that caused by the known constitutively-active ALK6 Q204D variant^35^. In addition to increased BRE-luciferase activity at baseline, the R371Q ALK6 variant increased relative luciferase activity, compared to wild-type ALK6, in the presence of BMP treatment (Fig. 6B). In HepG2 cells transfected with either R371Q ALK6 or wild-type ALK6, the R371Q ALK6 variant produced greater BMP-induced *DGAT2* mRNA levels than wild-type ALK6 (Fig. 6C,D). Similarly, in HepG2 cells transfected with either R371Q ALK6 or wild-type ALK6, the R371Q ALK6 variant resulted in increased levels of pSMAD1/5/8 and increased DGAT2 protein levels, as assessed by immunofluorescence (Fig. 6E,F). Western blot analyses of pSMAD1/5/8 and DGAT2 yielded similar results (Supplemental Fig. 12). Taken together, the results show that the R371Q variant in *ALK6* is a novel constitutively active and hyper-responsive receptor, which promotes increased BMP-induced *DGAT2* expression in HepG2 cells.

To further investigate the role of ALK6 in the regulation of DGAT2 expression, we used siRNA to decrease the level of ALK6 in HepG2 cells. Depletion of ALK6 inhibited the BMP2-induced increase in DGAT2 mRNA levels suggesting that BMP regulation of DGAT2 expression is dependent on ALK6 signaling (Supplemental Fig. 13).

## Discussion

This study identified a novel role of BMP signaling in non-alcoholic fatty liver disease. *In vivo*, BMP signaling was essential for the development of hepatic steatosis, independent of plasma cholesterol levels or glucose tolerance. *In vitro*, hepatocyte lipid accumulation and DGAT activity were induced by BMP signaling. Based on these findings, we performed a candidate gene analysis that identified a rare variant of *ALK6* associated with NAFLD in a human cohort. Functional studies of the *ALK6* variant revealed that it codes for a previously unknown, constitutively active form of the BMP receptor. The important role of BMP signaling in the pathogenesis of NAFLD suggests that the BMP signal transduction pathway may be a successful target of future approaches to the treatment of this disease.

BMP ligands play a role in the pathophysiology of inflammation and hepatic fibrosis, but their role in hepatic steatosis has not been established^19,21,36^. *In vitro*, upregulation of BMP9 enhanced damage to hepatocytes and promoted liver fibrosis, whereas *in vivo*, BMP7 was associated with reduced liver fibrosis^19–21^. In our experiments with *db/db* mice, BMP inhibition reduced markers of hepatic inflammation and demonstrated an important role for BMP signaling in the triglyceride accumulation associated with NAFLD. Other studies have implicated BMP signaling as a regulator of metabolism in non-hepatic tissues: Tseng *et al*. revealed the importance of BMP ligands in adipocyte energy metabolism and Graham *et al*. showed that fatty acid metabolism is altered in the right ventricle of BMPR2-deficient mice^37,38^. Of note, LDN-193189 inhibits all BMP type 1 receptors and is therefore nonselective^39^. ALK3-Fc, by contrast, is a soluble ligand trap that more potently inhibits BMP2 and BMP4 ligands with up to 50-fold lower affinity for BMP6 and BMP7^40^. Our data suggest that BMP2 and BMP4 may be particularly important in the development of NAFLD, given the effective inhibition of NAFLD by ALK3-Fc. Overall, the results from this study highlight the importance of BMP signaling in hepatic triglyceride metabolism.

BMP signaling influences the expression and activity of DGAT2, an important determinant of hepatic triglyceride levels. DGAT2 is tightly regulated in the liver and even small changes in expression result in significant changes in hepatic triglyceride levels in human genetic studies and multiple models of NAFLD^12–18^. In the present study, compared to vehicle-treated mice, *db/db* mice treated with either LDN-193189 or ALK3-Fc had reduced *Dgat2* expression. BMP inhibition also resulted in reduced hepatic steatosis without altering glucose tolerance, which is consistent with previous studies which showed that manipulation of *Dgat2* impacted hepatic steatosis without affecting glucose tolerance^13,17,41^. Our *in vitro* studies revealed that the BMP-DGAT2 relationship is preserved across three distinct hepatoma cell lines, and DGAT2 is regulated via the canonical (SMAD-dependent) BMP signaling pathway. This is the first report of BMP-mediated regulation of DGAT2 expression and activity.

Although multiple traditional genome-wide association studies of NAFLD have identified mild and moderate impact genetic variants, genome-wide association studies are limited to evaluating common genetic variants and, therefore, exclude rare genetic variants with higher functional significance^42–44^. We leveraged the clinical and genetic resources available at Vanderbilt University Medical Center to identify rs34970181 as a rare, but potent, contributor to NAFLD. Moreover, we determined the functional significance of this variant and demonstrated that the minor allele of this SNP codes for a constitutively active variant of the ALK6 receptor. Future studies are needed to replicate the association of rs34970181 and NAFLD. Two prior genome-wide association studies did not identify variants in BMP pathway genes that were associated with NAFLD. However, these studies excluded variants with a minor allele frequency less than 1% and would therefore not have included rs34970181^45,46^. The association of rs34970181 with NAFLD in humans supports the hypotheses that BMP signaling may be one of multiple “hits” which predispose an individual to NAFLD and that aberrant BMP signaling may be an important mechanism in a subset of NAFLD.

In summary, this study demonstrated that pharmacologic inhibition of the BMP signaling pathway prevented the development of hepatic steatosis in a murine model of NAFLD likely via SMAD-dependent regulation of Dgat2. A potential role for BMP signaling in human NAFLD was discovered with the identification of an association between a single nucleotide polymorphism in a BMP type 1 receptor and increased prevalence of NAFLD. Characterization of the functional implication of this variant revealed that it coded for a constitutively active ALK6 receptor that enhanced BMP-induced DGAT2 expression. Taken together, these in vivo, *in vitro*, and human genetic studies implicate BMP signaling in the pathogenesis of NAFLD and support the study of BMP inhibitors as potential treatments to alleviate the burden of this disease.

## Materials and Methods

### Animals and *in vivo* experiments

All experiments involving mice were approved by the Partners Subcommittee on Research Animal Care. Leptin receptor-deficient (*db/db*) mice and wild-type control mice on a C57BLKS/J background were purchased from Jackson Laboratories. All mice were male and were fed a standard chow diet. All animals received humane care according to the ARRIVE guidelines from the National Centre for the Replacement, Refinement and Reduction of Animals in Research as well as the criteria outlined in the “Guide for the Care and Use of Laboratory Animals” prepared by the National Academy of Sciences.

Mice were treated via intraperitoneal injection with LDN-193189 (0.33 or 1 mg/kg once daily), ALK3-Fc (2 mg/kg every other day), or vehicle alone beginning at four weeks of age. Vehicle for LDN-193189 was sterile water titrated to a pH of 5.5 with HCl to match the pH of the LDN-193189 solution. Vehicle for ALK3-Fc was TBS as recommended and obtained from the manufacturer of ALK3-Fc (Acceleron Pharma Inc.) and as similarly used in prior studies^20,40,47^. Experiments with LDN-193189 lasted two weeks and experiments with ALK3-Fc lasted three weeks. At the end of the studies, mice were sacrificed, plasma was obtained by cardiac puncture, and liver tissue was harvested for triglyceride quantification, qRT-PCR, histology, and Western blotting as described below and in the online supplement. Plasma markers of liver injury, alanine aminotransferase and alkaline phosphatase, total cholesterol, and triglyceride levels were measured by the Massachusetts General Hospital core laboratory.

Glucose tolerance testing was performed as described previously^48^. *db/db* mice were fasted overnight in wire-bottomed cages after 2 weeks of daily intraperitoneal injection with LDN-193189 (3 mg/kg daily) or vehicle. The following morning they were injected with LDN-193189 or vehicle two hours prior to an intraperitoneal glucose load (2 g/kg of 25% dextrose). A handheld glucometer was used to measure blood glucose in tail vein blood every 30 minutes for 150 minutes after administration of the glucose load.

### Histology

The right lobes of mouse livers were embedded and cryopreserved in “optimal cutting-temperature” medium (Sakura Tissue-Tek, Zoeterwoude, Netherlands), and 6-µm sections were prepared and fixed in situ with 10% formalin for histology using hematoxylin and eosin stain^36^.

### Triglyceride measurements

A colorimetric assay (Abcam, catalog #65336) was used to measure hepatic triglyceride levels, as directed by the manufacturer protocol. Briefly, liver lobes were weighed and ~50 mg of tissue was homogenized in 5% NP-40. After two cycles of heating the samples to 100 °C for 2 min followed by cooling to room temp, the samples were again heated and then microcentrifuged for 2 min at 16000 g. The soluble fraction was diluted 10 fold, combined with lipase and a proprietary reaction mix and incubated for 1 hour at room temperature prior to measuring the optical density at 570 nm.

### *In vitro* experiments

Hepatoma cell lines of human origin (HepG2, HuH7) primary human hepatocytes (Massachusetts General Hospital Cell Resource Core), and a murine hepatocyte cell line (AML12) were used to evaluate the effects of BMP signaling on DGAT2 expression *in vitro*. Cells were grown in EMEM with 10% fetal bovine serum. At the start of each experiment, cells were washed with PBS and incubated in serum-free media overnight. All *in vitro* experiments used a BMP ligand concentration of 20 ng/ml unless otherwise noted. An established *in vitro* model of hepatocyte triglyceride accumulation was used: HepG2 cells were incubated with 1.2 mM albumin-conjugated oleic acid (Sigma O3008) or 0.6 mM bovine serum albumin control (Fatty Acid-Free Protease Free Essentially Globulin Free, Sigma A7030) for 4 or 24 hrs^49,50^. Cells were subsequently harvested to isolate RNA. In additional experiments, cells were stimulated with BMP2 and harvested at 24hrs for RNA or protein. Primers used for qRT-PCR analyses are listed in Supplemental Table 1. All hepatic cell lines were purchased from the ATCC organization. Knockdown experiments are further described in the supplemental methods.

### Oil Red O quantification *in vitro*

For quantification of intracellular neutral lipid accumulation, Oil Red O staining and quantification was carried out as previously described^51^. Briefly, after 24h of incubation with oleic acid in the presence or absence of BMP2 and/or LDN-193189, HepG2 cells were washed x3 with cold PBS and fixed with 10% formalin for 1 hour. Next, they were stained with freshly prepared Oil Red O working solution (Sigma C0625) for 60 min. Excess Oil Red O solution was washed off of cell plates with water. The remaining Oil Red O dye was solubilized by isopropanol and quantified spectrophotometrically at 510 nm. Background signal was determined by assessing Oil Red O in cells treated with BSA instead of oleic acid.

### *In vitro* DGAT activity assay

HepG2 cells starved overnight were treated with either vehicle or BMP2 (20 ng/mL) for 24 hours. DGAT2 activity assay was performed as previously described^52^. Please refer to online methods supplement for further details.

### Chromatin immunoprecipitation combined with qPCR

ChIP-qPCR was carried out as previously described on cells treated with either DMSO, BMP2 (20 ng/mL), LDN (100 nM), or IgG for 48hrs^53^. Please refer to online methods supplement for further details.

### BMP-responsive luciferase reporter assay

C2C12BRA cells were stably transfected with a BMP-responsive, Id1 promoter, firefly luciferase reporter gene construct to assess BMP signaling, as previously described^54,55^. Lipofectamine (Invitrogen) was used to transfect cells containing the reporter gene with wild-type ALK6, rs34970181 (R371Q) ALK6, or constitutively active (Q204D) ALK6 (kindly provided by Dr. Takeshi Imamura, JFCR Cancer Institute, Tokyo, Japan). Transfection efficiency was determined using pRL-TK renilla luciferase (Promega). The ratio of firefly to renilla luciferase activities was calculated to quantify the amount of BMP signaling normalized to transfection efficiency. For BMP stimulation experiments, transfected C2C12BRA cells were incubated for 16 hours in serum-free media together with 0, 5, or 25 ng/ml of BMP2.

### Approach for association analysis of variants in the BMP type 1 receptors and NAFLD

The Vanderbilt University Medical Center DNA biorepository BioVU and a de-identified version of the electronic health record were used to test for an association between NAFLD and coding genetic variants in BMP type 1 receptor genes (*ALK2*, *ALK3*, and *ALK6*)^56^. First, SNPs in BMP type 1 receptor genes were subjected to a screening analysis with the previously established phenotype PheCode 571.5 which codes for all non-infectious and non-alcoholic liver disease (including, but not limited to, NAFLD). Please refer to the online supplemental methods for further details. Thereafter, the top SNP of interest was directly evaluated for association with NAFLD by a retrospective cohort study.

### A retrospective cohort analysis of rs34970181 carriers and matched controls for association with NAFLD

Because the ICD-9 coding system did not include a specific code for NAFLD and the use of diagnostic billing codes alone could result in incomplete and/or inaccurate ascertainment of NAFLD, we performed a cohort analysis comparing the prevalence of NAFLD in minor allele carriers of rs34970181 and non-carriers as determined by manual review of de-identified electronic health records. rs34970181_A minor allele carriers were matched in a 1:2 ratio to non-carrier controls (N = 144) by age (+/−1 year), sex, race, and BMI. For each individual, all radiology reports were queried for “steatosis”, “NAFLD”, “steatohepatitis”, “liver”, and “hepatic”. Review of each chart was conducted by a physician blinded to SNP carrier status. Hepatic steatosis was determined by any radiology report explicitly affirming or denying the presence of hepatic steatosis or pseudonym, (i.e. “fatty liver”, “steatohepatitis”). In the case of conflicting radiology reports of hepatic steatosis for a single individual, the individual was counted as having hepatic steatosis. Additionally, pathology reports were reviewed for biopsy-proven disease. The medical records of patients with hepatic steatosis by imaging or pathology were further reviewed to exclude non-NAFLD causes of hepatic steatosis: excessive alcohol consumption, hepatitis C, parenteral nutrition, pregnancy, and medications (amiodarone, methotrexate, tamoxifen, valproate, and anti-retrovirals). Documented ongoing daily alcohol intake exceeding 10 g per day for women or 20 g per day for men were considered to be alcohol-related fatty liver disease, as previously defined^57^. Diabetes mellitus status was determined by at least 2 instances of diabetes mellitus ICD diagnostic codes. All provided lab values in Table 2 are means of the median captured values for each individual.

### Statistical analysis

Statistical analyses were performed using GraphPad Prism 5.0 (GraphPad Software, La Jolla, CA). Data are reported as mean ± SEM, unless otherwise indicated. Two group comparisons of continuous variables were performed using the two-tailed Student’s t test. For more than 2 group comparisons of continuous variables, analysis of variance (ANOVA) with post-hoc testing corrected for multiple comparisons was employed. For human categorical data, including NAFLD and diabetes status, Fisher’s exact test was used to make comparisons. In all cases, P < 0.05 was considered to indicate statistical significance.

### Study approval

#### All experiments were performed in accordance with relevant guidelines and regulations

Mouse studies were carried out in strict accordance with the recommendations in the Guide for the Care and Use of Laboratory Animals of the National Institutes of Health. Housing and all procedures involving mice described in this study were specifically approved by the Institutional Animal Care and Use Committees of Massachusetts General Hospital (Subcommittee on Research Animal Care, protocols #2012N000166 and #2008N000169).

IRB approval at Vanderbilt University Medical Center was obtained for access and analysis of de-identified electronic medical record data and archived genetic information (“Leveraging PheWAS for Drug Repurposing” PI Jill Pulley IRB# 151121). Informed consent was obtained from all participants.

## Supplementary information

Supplemental methods, figures, and tables.
