# Supplementary material for: The Role of Bone Morphogenetic Protein Signaling in Non-Alcoholic Fatty Liver Disease
Source: Sci Rep. 2020 Jun 19;10:9831. doi: 10.1038/s41598-020-66770-8 (PMC7305229; doi:10.1038/s41598-020-66770-8)
Supplement: Supplementary file 1 — Supplemental methods, figures, and tables. [file 41598_2020_66770_MOESM1_ESM.pdf]

Supplementary data

The Role of Bone Morphogenetic Protein Signaling in Non-Alcoholic Fatty Liver Disease

Timothy E. Thayer[1, 6], Christian L. Lino Cardenas[1], Trejeeve Martyn[1], Christopher J Nicholson[1], Lisa Traeger[2], Florian Wunderer[2], Charles Slocum[1], Haakon Sigurslid,[1] Hannah R. Shakartzi[2], Caitlin O'Rourke[2], Georgia Shelton[1], Mary D. Buswell[1], Hanna Barnes[1], Leif R Neitzel[7], Clara D. Ledsky[2], Pingcheng Li[2], Megan F. Burke[1], Eric Farber-Eger[6], Daniel S. Perrien[6], Ravindra Kumar[5], Kathleen E. Corey[3,] Quinn S. Wells[6], Kenneth D. Bloch[1,2t], Charles C. Hong[7], Donald B. Bloch[#2,4], Rajeev Malhotra[#*1]

[1]Cardiovascular Research Center and Cardiology Division of the Department of Medicine; [2]Anesthesia Center for Critical Care Research of the Department of Anesthesia, Critical Care, and Pain Medicine; [3]GI Unit; and [4]Center for Immunology and Inflammatory Diseases and the Division of Rheumatology, Allergy, and Immunology of the Department of Medicine, Massachusetts General Hospital, Harvard Medical School, Boston, MA, United States

[5]Acceleron Pharma, Inc. Cambridge, MA, United States

[6]Department of Medicine, Vanderbilt University Medical Center, Nashville, TN, United States

[7]Department of Medicine, University of Maryland School of Medicine, Baltimore, MD, United States

[t] Deceased

[#] Authors contributed equally to this manuscript

*Corresponding author

Supplemental Methods

Chemicals and reagents

LDN-193189 (4-[6-(4-piperazin-1-ylphenyl)pyrazolo[1,5-a]pyrimidin-3-yl]quinoline) was synthesized as previously described,[1] dissolved in sterile water at a concentration of 0.5 mg/mL, and titrated to a pH of 5.5. Recombinant ALK3-Fc was provided by Acceleron Pharma Inc. (Cambridge, MA). pH-matched diluent-only solutions were used as controls in experiments using LDN-193189 and ALK3-Fc.

Measurement of gene expression by quantitative RT-PCR

Total RNA from livers and cultured cells was extracted by the phenol/guanidinium method.[2] Reverse transcription was performed using Moloney murine leukemia virus reverse transcriptase (Promega, Madison, WI, USA) or High-Capacity cDNA Reverse Transcription Kit (Applied Biosystems, Foster City, CA, USA). A Mastercycler ep Realplex (Eppendorf, Hamburg, Germany) was used for real-time amplification and quantification of transcripts. Relative expression of target transcripts were normalized to levels of 18S ribosomal RNA, determined using the relative C_T method. Taqman® gene expression assays were used to quantify *ID1* and *DGAT2* mRNA levels for both human and murine samples. Quantitative RT-PCR was performed with SYBR green for *18s* and additional genes, using the primer sequences shown in Supplemental Table 1.

Immunoblot techniques

Livers and cultured cells were homogenized in RIPA buffer containing protease and phosphatase inhibitors (Sigma). Tissue lysates (20 µg/lane) were separated by SDS-PAGE, transferred to

polyvinylidene difluoride membranes (GE Amersham Biosciences), and probed with antibodies specific for phosphorylated SMAD1/5/8 (P-SMAD1/5/8, catalog # D6656, Main Medical Center Research Institute), total SMAD 1 (Life Span, catalog #C75853), vinculin (H-10, sc-25336), DGAT2 (Santa Cruz, catalog #66859), HA Epitope Tag (Invitrogen, catalog #26183) or glyceraldehyde 3-phosphate dehydrogenase (GAPDH, Cell Signaling, catalog #2118L). Blots were incubated with horseradish peroxidase-conjugated anti-rabbit or anti-mouse IgG or fluorescent dye labeled anti-rabbit IgG IRDye 800CW (LI-COR, Lincoln, NE). Bound secondary antibodies were visualized either by chemiluminescence (ECL Plus) and quantified using a VersaDoc Imaging System (BioRad, Hercules, CA) or using a LI-COR Odyssey detection system (LI-COR, Lincoln, NE).

Liver slides were used for immunofluorescence staining of IL-1b (sc-12742) and Mmp9 (sc-21733). Primary antibodies were incubated for 2 hrs at room temperature followed by 3 washes with PBS-Tween20 (0.1%). Then secondary antibodies were incubated for 45min followed by 4 washes and slides were mounted with fluoroshield mounting medium with DAPI (ab104139). Slides were visualized with the Leica TCS SP8 confocal microscopy station and pictures were digitized with the Leica Application Suite X software.

HepG2 cells were rinsed twice with cold PBS and proteins were extracted using RIPA buffer supplied with Halt Protease and Phosphatase Inhibitor Cocktail-EDTA (1x). Total protein concentration was determined by the colorimetric bicinchoninic acid assay (BCA assay, Thermo Fisher). 20 µg of total protein from cell lysates were separated by SDS-PAGE and transferred onto 0.45 µm nitrocellulose membranes for Dgat2 protein detection. Briefly, primary antibody was incubated over night at 4^0C and then membranes were washed twice followed by incubation with secondary antibody for 1 hr at room temperature. SuperSignal West Pico chemiluminescent

substrate (Thermo Fisher) was added to the membranes. Bands were visualized with the C-DiGit blot scanner (Licor Technologies). pSMAD 1/5 antibody was obtained from Main Medical Center Research Institute (Catalog # D6656/Vli31).

Chromatin immunoprecipitation combined with qPCR (ChIP-qPCR)

Ten million HepG2 cells were treated with DMSO, recombinant BMP2 (20 ng/mL), BMP signaling inhibitor (LDN-100nM), or IgG for 48 hrs. ChIP-qPCR was carried out as previously described.[3] Cells were fixed and prepared for ChIP-qPCR using the EpiTect ChIP kit, according to the manufacturer's instructions (Qiagen, USA). Briefly, 50 µg of total protein lysates were sonicated to shear chromatin to an average length of 500-1500 bp followed by centrifugation for 10 min. Supernatants were collected in 2mL tube containing 6µg of monoclonal antibodies against SMAD1/5 (Abcam, AF10B7) followed by overnight incubation at 4^0C. qPCR primers used to scan the promoter of DGAT2 were F: 5'CTACTAAAGCTCTCCCAAG3' AND R: 5'GGAATTTTGCAGATGGAAG3'. Equal amounts of DNA (32ng) was used to perform the qPCR using SYBR green system (Applied Biosystem, Foster City, CA).

In vitro DGAT activity assay

HepG2 cells starved overnight were treated with either vehicle or BMP2 (20 ng/mL) for 24 hours. As a positive control, cells were transfected with a pCMV3-C-FLAG DGAT2 plasmid (Sino Biological, Catalog #HG14114-CF) or empty pCMV3-C FLAG. Fractionation of membrane-bound proteins was performed using a subcellular proteome extraction kit (Roche, Catalog #539790), according to the manufacturer's instructions. Protein concentrations were determined per manufacturer's protocol (Bio-Rad Laboratories, Catalog #500-0119). DGAT

activity assays were performed with fluorescently-labeled palmitoyl CoA, as previously described.[4] Briefly, 150 µL of reaction mix containing 33 µM N-[(7-nitro-2-1,3-benzoxadiazol-4-yl)-methyl]amino-(NBD)-palmitoyl CoA, 27 mM $MgCl_2$, 270 µM 1,2 dioleoyl-sn-glycerol (DOG) in acetone (Sigma, Catalog #D0138), and 0.8 mg/mL BSA solution in 130 mM Tris-HCl (pH 7.6) was added to 50 µg of each protein sample, for a total reaction volume of 200 µL. After five minutes at 37°C, the reaction was terminated with chloroform/methanol (2:1 v/v). The samples remained at room temperature for one hour and were then centrifuged for five minutes at 3000 rpm. The aqueous top layer was aspirated off from each sample, and the remaining organic layer was dried using a stream of nitrogen gas. The dried reaction products were resuspended in chloroform/methanol and spotted on a 20 x 20 cm channeled TLC plate (Sorbent Technologies, Catalog #2315126C) for separation. The solvent system consisted of hexane/ethyl ether/acetic acid (80:20:1, v/v/v), as previously described. The plate was imaged at the appropriate wavelength (the excitation and emission wavelengths for NBD are 465 nm and 535 nm, respectively). Quantification of NBD fluorescence was conducted using ImageJ software. Data are presented as normalized units of fluorescent intensity per minute per µg protein.

ALK6 variant plasmid construction

The rs34970181 minor allele construct was produced using a cDNA encoding wild-type ALK6 kindly provided by Dr. Takenobu Katagiri (JFCR Cancer Institute, Tokyo, Japan).[5] The Q5 site-directed mutagenesis kit (Qiagen) and the following oligonucleotides were used to introduce the R371Q polymorphism into the wild-type cDNA: R371Q_F, CCCAACACCCAGGTTGGCACC; and R371Q_R: TGGGATGTCAACCTCATTTGTG. Full plasmid contruct sequencing available as separate supplemental file.

Depletion of SMADs using siRNA

siRNAs targeting *SMAD1* and *SMAD5* (siSMAD) and scrambled control siRNA (siCTRL) were obtained from Dharmacon (SMARTpool, Thermo Scientific). HepG2 cells were transfected with siRNA using Lipofectamine RNAiMAX reagent, as described by the manufacturer (Life Technologies). Successful knockdown of SMAD1 and SMAD5 was confirmed by harvesting cells for protein and assessing SMAD levels by immunoblot. In parallel wells of the same experiments, cells were harvested to assess the change in *ID1* and *DGAT2* mRNA levels in response to BMP2 stimulation.

In a subset of experiments, HepG2 cells were treated with siRNA targeted to BMPR1B (ALK6) for 24 hours. Following serum starvation for 24 hours, cells were treated for a further 24 hours with BMP2 ligand and harvested to isolate RNA. siRNA directed against BMPR1B (siALK6) and scrambled control siRNA (siCTRL) were obtained from Dharmacon (SMARTpool, Thermo Scientific). Cells were transfected with siRNA using Lipofectamine RNAiMAX reagent, as described by the manufacturer (Life Technologies).

Screening analysis for identification of missense variants in BMP type 1 receptors associated with non-alcoholic and non-infectious liver disease

The screening analyses of missense variants in BMP type 1 receptors were performed by logistic regression of carrier status of minor alleles and presence of the PheCode 571.5. These analyses were performed using the BioVU Illumina exome chip cohort which includes 20,224 adults of European descent whose genotypes were determined using the Illumina HumanExome BeadChip

array v.1.0 as part of several BioVU research initiatives. This chip contains ~250,000 single nucleotide polymorphisms (SNPs), including 17 missense variants in ALK2, ALK3, and ALK6.[6-8] A liberal minor allele frequency cutoff of >0.05% was set with the intention of functionally validating any missense SNPs of interest. The missense variants that met criteria were tested for association by logistic regression with the PheCode 571.5 which incorporates ICD-9 codes 571.8 and 571.9 which capture coding for non-alcoholic and non-infectious liver diseases (performed on 09/18/2016). Using PheCodes as phenotypes has been established as a validated method for identifying gene-disease association.[7] Each SNP was tested individually using logistic regression with an additive model containing the covariates of age and sex. Bonferroni correction was applied to account for multiple testing. Analyses were restricted to individuals of white European descent to limit population stratification.[9,10] The top SNP of interest emerging from this screening analysis was selected for specific testing for association with NAFLD.

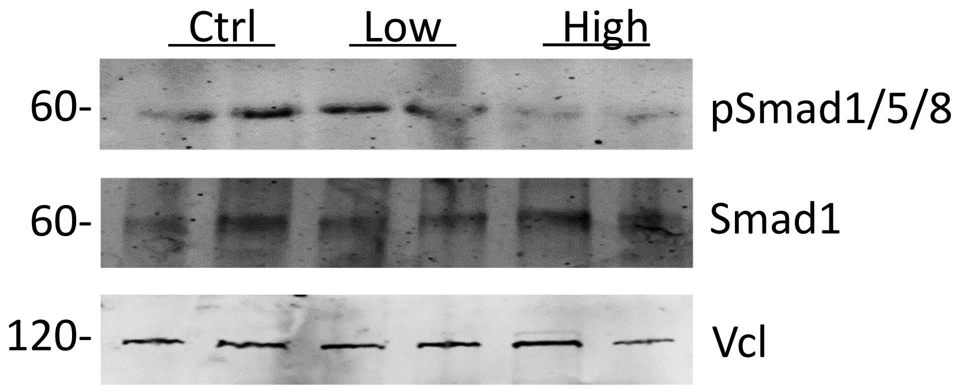

Supplemental Figure 1: BMP signaling inhibition was achieved in *db/db* mice with low (0.33mg/kg/d) and high-dose (1mg/kg/d) LDN-193189 treatment compared to vehicle-treated mice as assessed by phosphorylated Smad 1/5/8 levels.

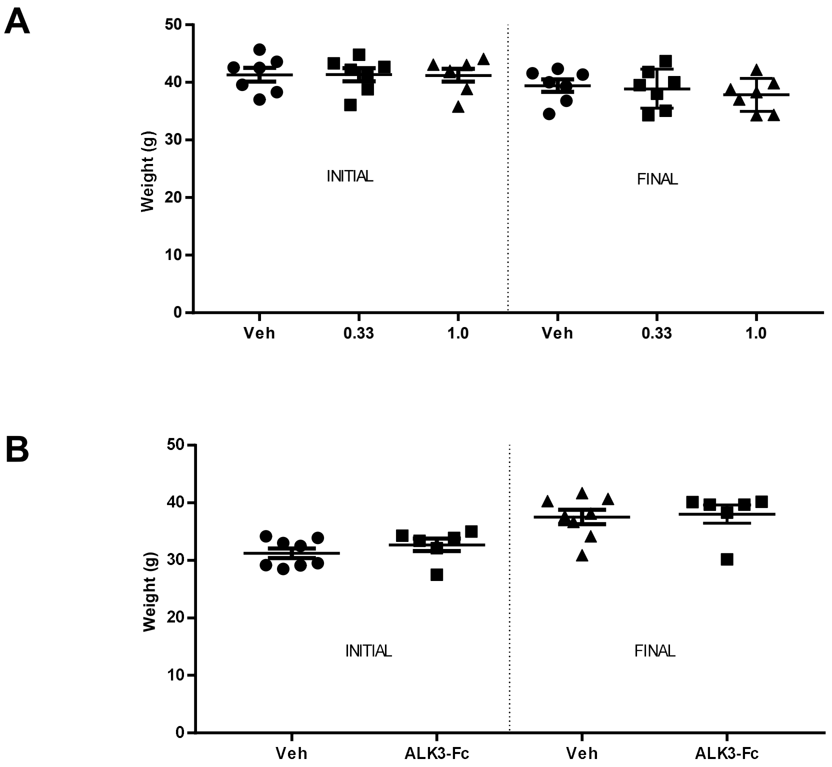

Supplemental Figure 2: There was no difference in initial or final weights in mice treated with BMP inhibitors or vehicle. Initial and final weights for mice treated with vehicle or LDN-193189, at one of two doses (0.33mg/kg daily or 1.0 mg/kg daily, A). Initial and final weights for mice treated with vehicle or ALK3-Fc every other day (B). Comparisons were performed using 1-way ANOVA and two-tailed Student's t test respectfully.

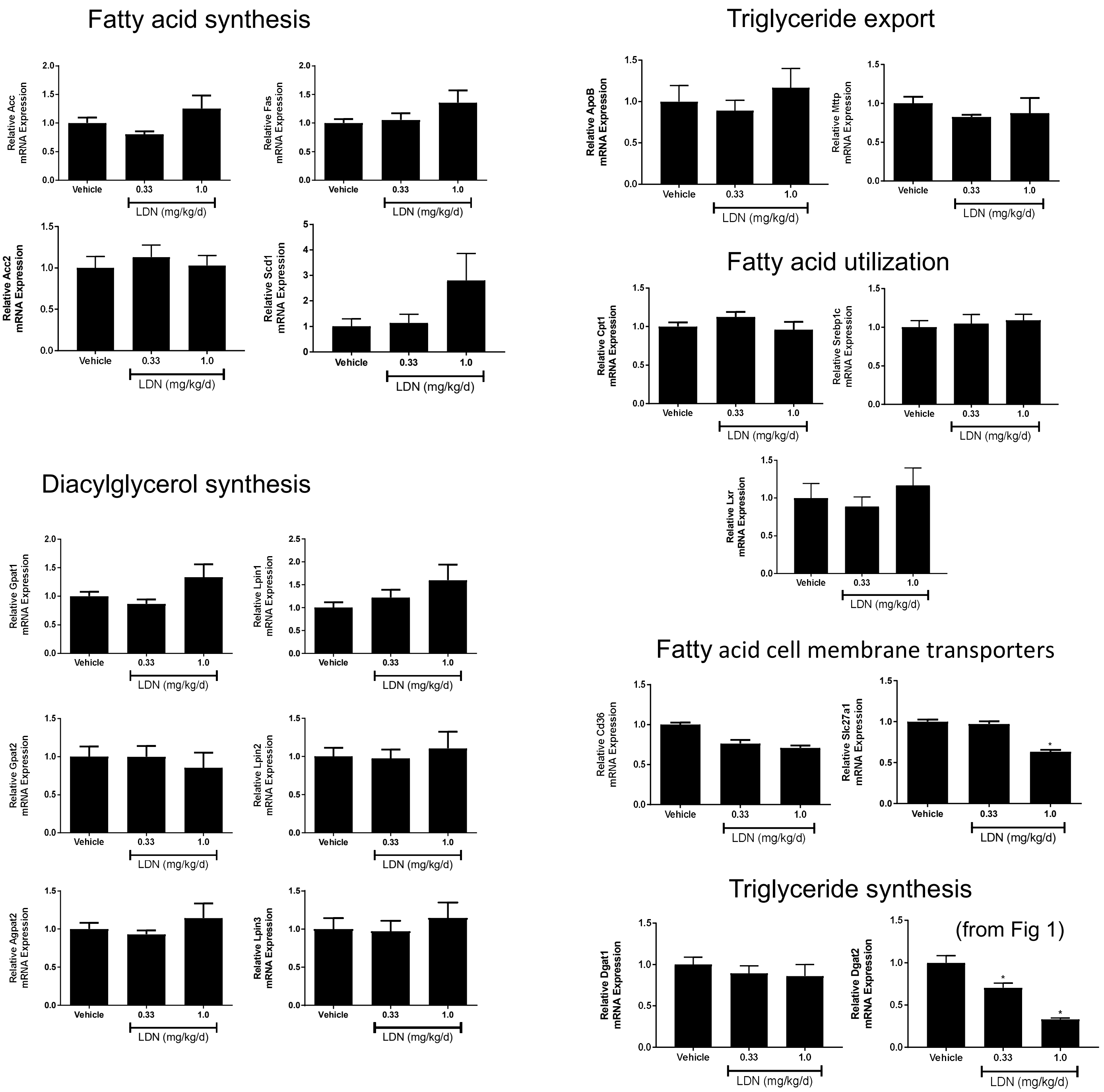

Supplemental Figure 3: BMP signaling inhibition with LDN-193189 did not significantly alter the hepatic mRNA expression of key lipid metabolism and triglyceride synthesis genes other than *Dgat2* in *db/db* mice. qRT-PCR for expression of genes in mice treated for two weeks with vehicle or LDN-193189. All mRNA levels were normalized to 18S RNA levels. There were seven replicates per treatment group. Comparisons were performed using 1-way ANOVA with Sidak's multiple comparison testing.

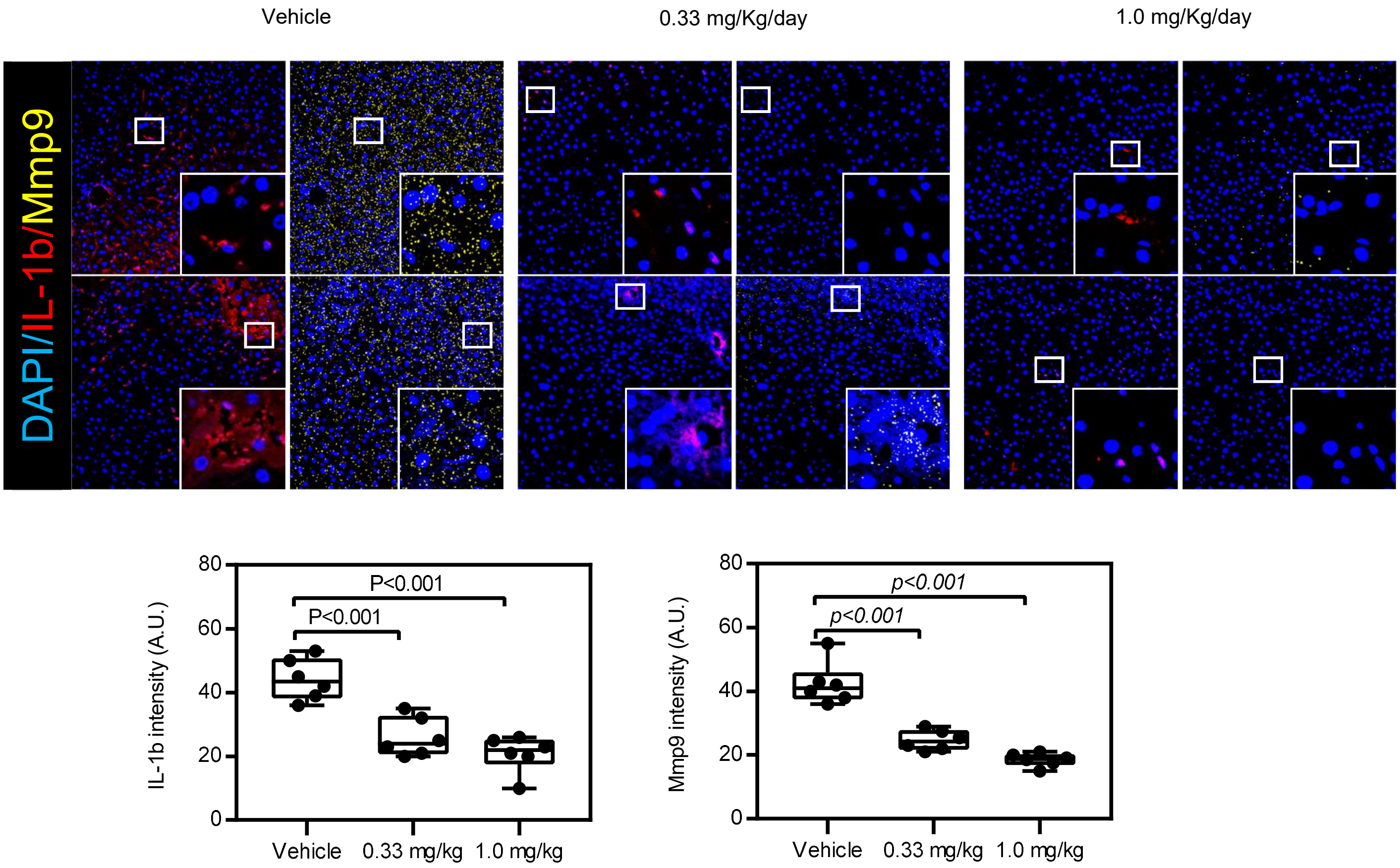

Supplemental Figure 4: Pharmacologic inhibition of BMP signaling with LDN-193189 reduces hepatic mediators of inflammation and fibrosis. Liver sections from *db/db* mice treated with LDN-193189 were stained for interleukin 1-beta (IL-1b, in red), matrix metalloproteinase-9 (Mmp9, in yellow), or DAPI (in blue). Comparisons were performed using 1-way ANOVA with Sidak's multiple comparison testing.

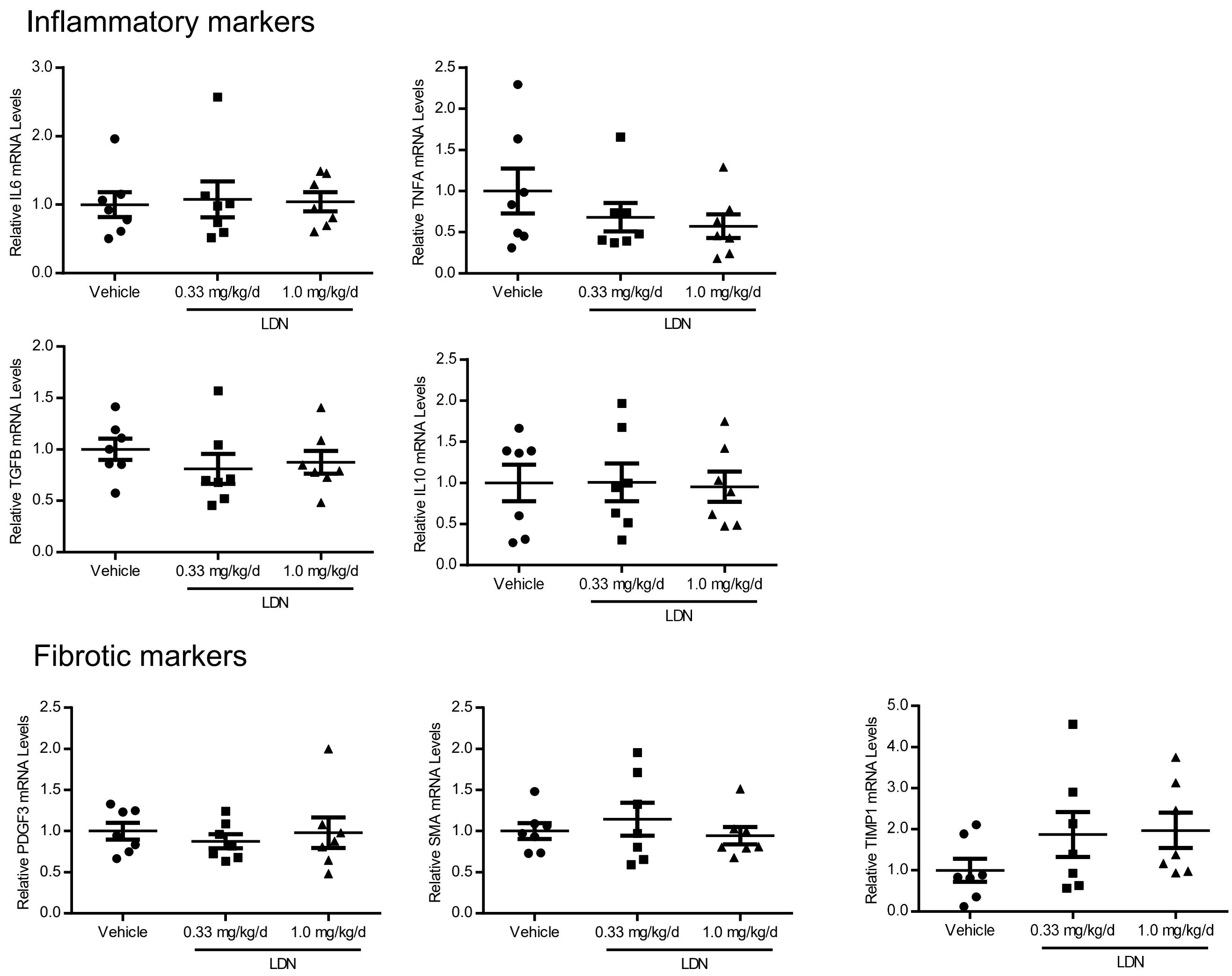

Supplemental Figure 5: BMP signaling inhibition with LDN-193189 did not alter the hepatic mRNA expression of inflammatory and fibrotic genes in *db/db* mice. qRT-PCR for expression of genes in mice treated for two weeks with vehicle or LDN-193189. All mRNA levels were normalized to 18S RNA levels. There were seven replicates per treatment group. Comparisons were performed using 1-way ANOVA with Sidak's multiple comparison testing.

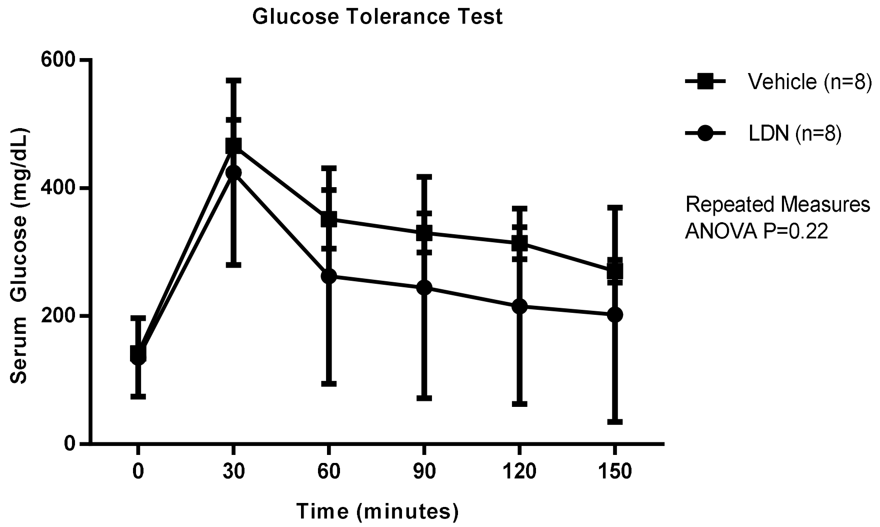

Supplemental Figure 6: Treatment of db/db mice with LDN-193189 did not alter glucose tolerance compared to vehicle-treated mice. Mice were treated for two weeks with vehicle control or LDN-193189 prior to glucose tolerance testing.

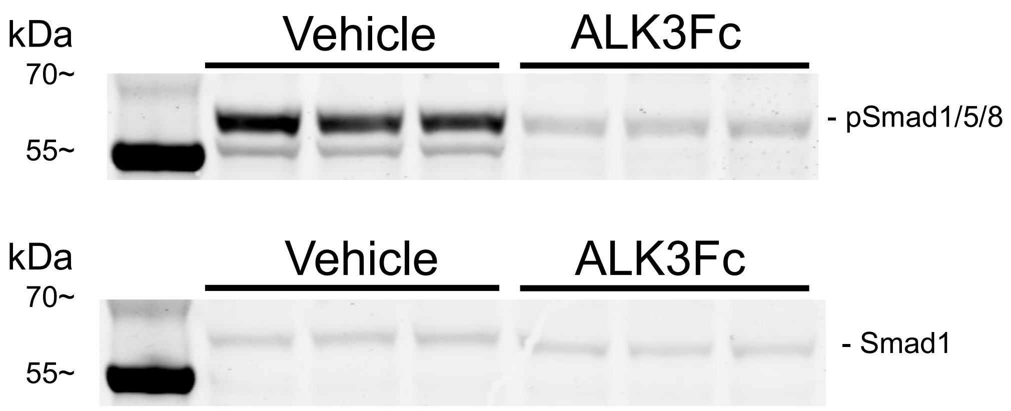

Supplemental Figure 7: BMP signaling inhibition was achieved in *db/db* mice with ALK3Fc treatment compared to vehicle-treated mice as assessed by phosphorylated Smad 1/5/8 levels

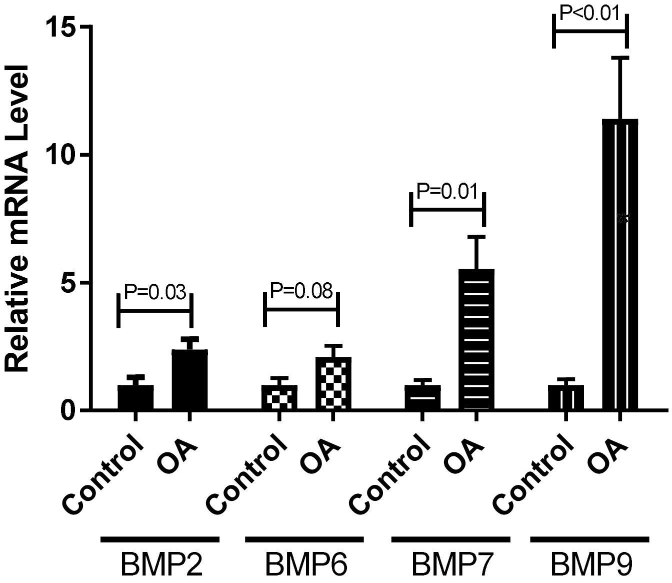

Supplemental Figure 8: Treatment of HepG2 cells with oleic acid (OA) or vehicle control resulted in increased mRNA expression of BMP 2, 7, and 9 at 24 hours. Comparisons performed using 2-tailed Student's t test.

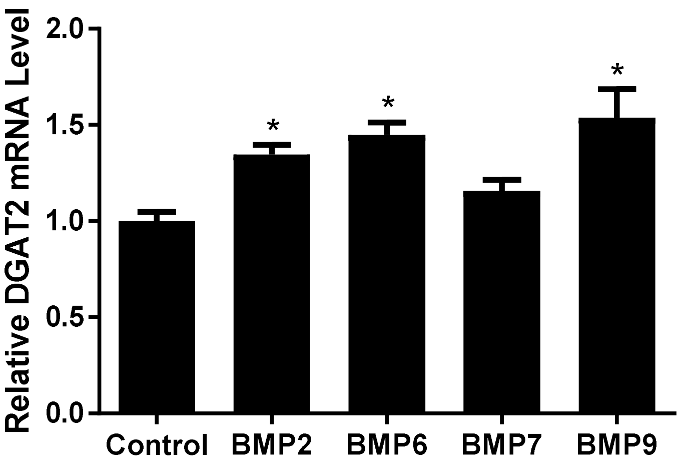

Supplemental Figure 9: The effect of stimulation of HepG2 cells with various BMP ligands *in vitro* on mRNA expression of *DGAT2*. Comparisons performed using 1-way ANOVA with Sidak's post-test for multiple comparisons (* $p<0.05$ compared to control).

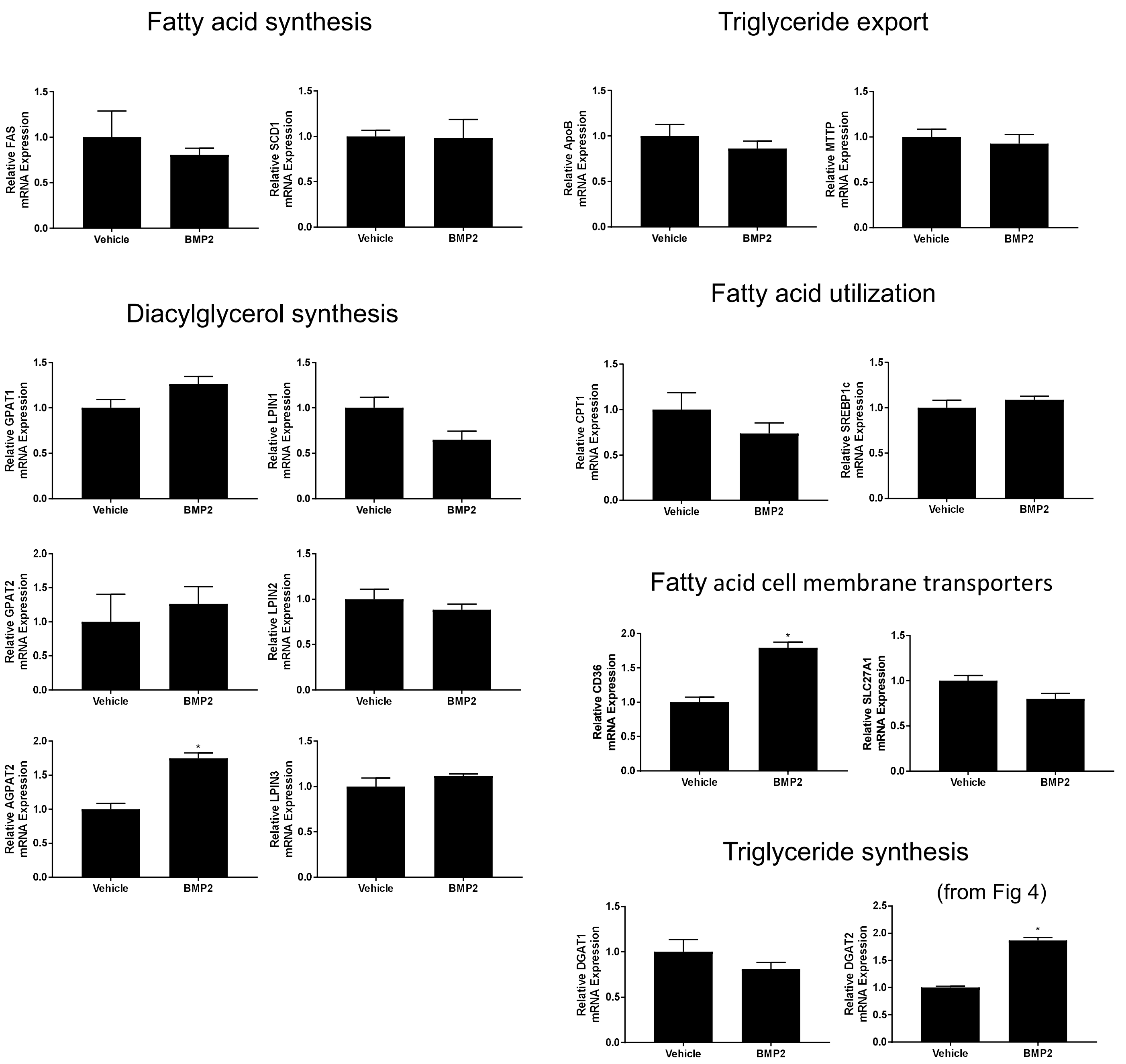

Supplemental Figure 10: The effect of BMP2 stimulation *in vitro* on mRNA expression of key lipid metabolism genes. qRT-PCR was performed to determine mRNA levels of key proteins involved in lipid metabolism in HepG2 cells treated with vehicle or BMP2 for 24 hours. All expression levels were normalized to 18S RNA levels. Comparisons were made using the two-tailed Student's t test.

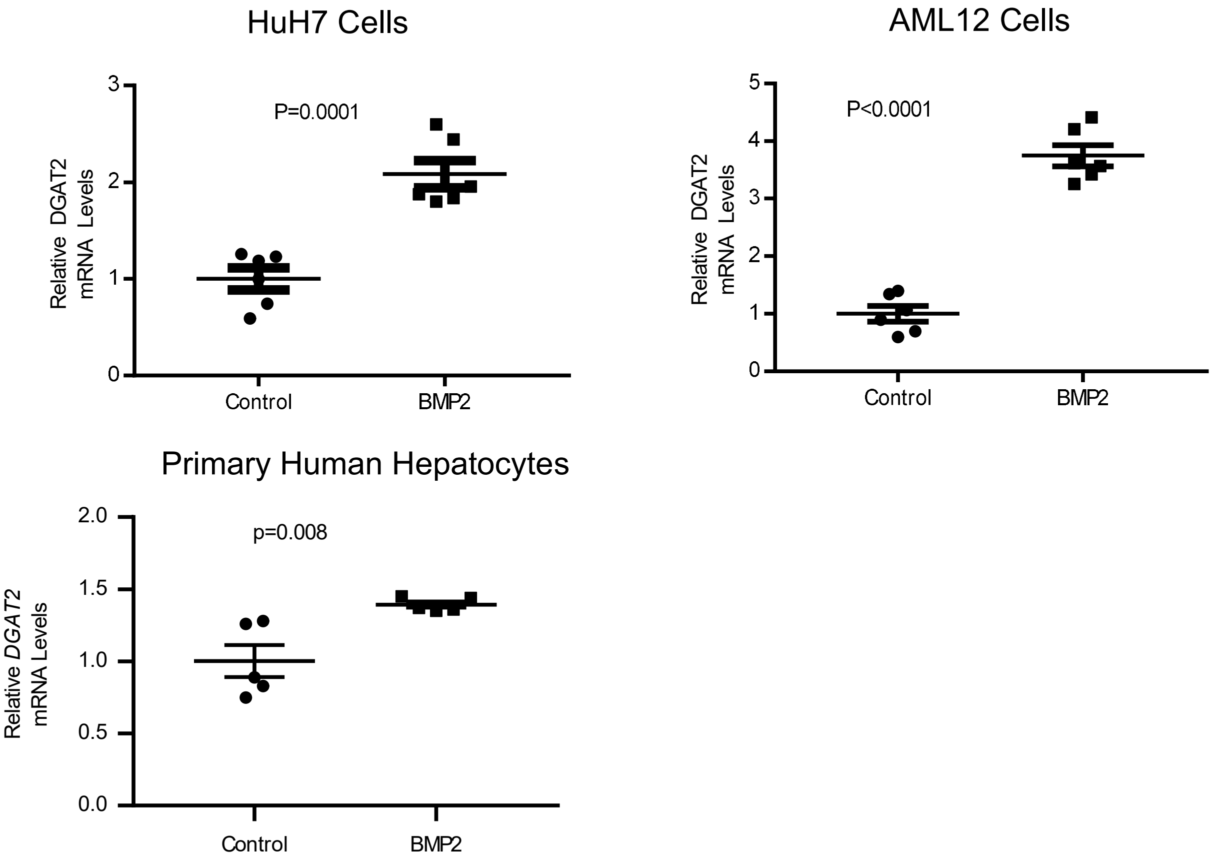

Supplemental Figure 11: BMP signaling induced DGAT2 in multiple cell lines. qRT-PCR for *DGAT2* mRNA expression was performed in cells treated with vehicle or BMP2 for 24 hours. All expression levels were normalized to 18S RNA levels. Comparisons were made using the two-tailed Student's t test.

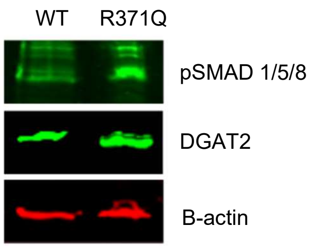

Supplemental Figure 12: Western blot for pSMAD1/5/8 and DGAT2 from HepG2 cells transfected with wild-type ALK6 or R371Q ALK6.

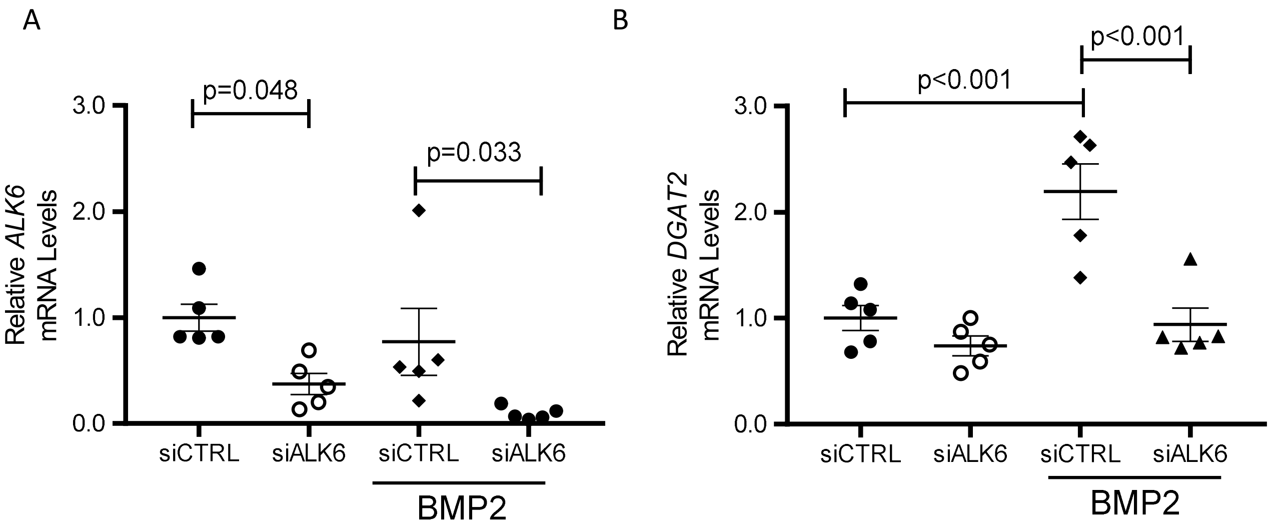

Supplemental Figure 13: BMP2-induced DGAT2 expression is dependent on ALK6. Targeted siRNA knockdown of ALK6 in HepG2 cells was performed (A). BMP2 induction of DGAT2 was precluded by ALK6 knockdown (B). Five replicates per group. Comparisons were performed using 1-way ANOVA with Sidak's multiple comparison testing.

Supplementary Table 1: Primer sequences utilized for gene expression analysis.

Gene Name	Forward Primer	Reverse Primer
18S(human,murine)	5’-CGGCTACCACATCCAAGGAA-3’	5’-GCTGGAATTACCGCGGCT-3’
Acc(murine)	5’-CTCCCGATTCATAATTGGGTCTG-3’	5’-TCGACCTTGTTTTACTAGGTGC-3’
Acc2(murine)	5’-CGCTCACCAACAGTAAGGTGG -3’	5’-GCTTGGCAGGGAGTTCCTC-3’
Agpat2(murine)	5’-CAGCCAGGTTCTACGCCAAG-3’	5’-TGATGCTCATGTTATCCACGGT-3’
ApoB(murine)	5’-GCTCAACTCAGGTTACCGTGA-3’	5’-AGGGTGTACTGGCAAGTTTGG-3’
Cpt1(murine)	5’-TGGCATCATCACTGGTGTGTT-3’	5’-GTCTAGGGTCCGATTGATCTTTG-3’
Dgat1(murine)	5’-GTGCCATCGTCTGCAAGATTC-3’	5’-GCATCACCACACACCAATTCAG-3’
Fas(murine)	5’-GCGGGTTCGTGAAACTGATAA-3’	5’-GCAAAATGGGCCTCCTTGATA-3’
Gpat1(murine)	5’-CTTGGCCGATGTAAACACACC-3’	5’-CTTCCGGCTCATAAGGCTCTC-3’
Gpat2(murine)	5’-AGCAGAGGAGTAACCACAATGG-3’	5’-AGCAGAGGAGTAACCACAATGG-3’
Lpin1(murine)	5’-CCTCCGCTCCCGAGAGAAA-3’	5’-CGTTGTCTCCCAACTTCATGT-3’
Lpin2(murine)	5’-CAGAGTTCAGACGTTTCTCACAC-3’	5’-GCTCCTTGTAGTCCTCTTCCT-3’
Lpin3(murine)	5’-CAAACCTCGTGGTGAAAATCAAC-3’	5’-CCACAGTGCTCTCAGGTAAGT-3’
Lxr(murine)	5’-CTGATTCTGCAACGGAGTTGT-3’	5’-GACGAAGCTCTGTCGGCTC-3’
Mttp(murine)	5’-ATACAAGCTCACGTACTCCACT-3’	5’-TCTCTGTTGACCCGCATTTTC-3’
Scd1(murine)	5’-TTCTTGCGATACACTCTGGTGC-3’	5’-CGGGATTGAATGTTCTTGTCGT-3’
Srebp1c(murine)	5’-AACGTCACTTCCAGCTAGAC-3’	5’-CCACTAAGGTGCCTACAGAGC-3’
AGPAT2(human)	5’-GCCGAGTTCTACGCCAAGG-3’	5’-CGAACCAGCCGATGATGCT-3’
APOB(human)	5’-GAAGCAGGTTTTCCTTTACCCG-3’	5’-GCCCCTCTTGATGTTCAGGATG-3’
CPT1(human)	5’-TCCAGTTGGCTTATCGTGGTG-3’	5’-TCCAGAGTCCGATTGATTTTTGC-3’
DGAT1(human)	5’-TATTGCGGCCAATGTCTTTGC-3’	5’-CACTGGAGTGATAGACTCAACCA-3’
FAS(human)	5’-TCTGGTTCTTACGTCTGTTGC-3’	5’-GGGCATTAACACTTTTGGACGAT-3’
GPAT1(human)	5’-TCTTTGGGTTTGCGGAATGTT-3’	5’-ATGCACATCTCGCTCTTGAATAA-3’
GPAT2(human)	5’-TGCACCGTGTGACATAGACC-3’	5’-GCACTGACGATGTATTCCTGC-3’
LPIN1(human)	5’-GAAGTTATCCCTATGCACCTGGC-3’	5’-GCTGGGAGCGATCACTTGG-3’
LPIN2(human)	5’-TCTACAAGGGCATTAACCAGGC-3’	5’-AACGTGAAAAGGTGAACACTGA-3’
LPIN3(human)	5’-GGAGGAAACCCAAGCAGAAAG-3’	5’-AGGGATAGCTCACTCTCAGCG-3’
MTTP(human)	5’-ACAAGCTCACGTACTCCACTG-3’	5’-TCCTCCATAGTAAGGCCACATC-3’
SCD1(human)	5’-TCTAGCTCCTATACCACCACCA-3’	5’-TCGTCTCCAACTTATCTCCTCC-3’
SREBP1c(human)	5’-ACAGTGACTTCCCTGGCCTAT-3’	5’-GCATGGACGGGTACATCTTCAA-3’

Supplementary Table 2. Association analysis of BMP type 1 receptor polymorphisms and non-alcoholic chronic liver disease

Six missense SNPs in BMP type 1 receptor genes were available for analysis. Carrier status for each allele was assessed for association with the PheWAS code 571.5, which codes for non-alcoholic and non-infectious liver disease including, but not limited to, NAFLD.

BMP Type 1 Receptor	rsID	Gene	MAF	OR	P value	minor carrier cases	minor carrier controls	non-carrier cases	non-carrier controls
ALK2	rs34056189_C	ACVR1	0.00069	0	0.94	0	32	723	19469
ALK2	rs13406336_C	ACVR1	0.008111	0.57	0.14	7	331	715	19165
ALK3	rs35619497_T	BMPR1A	0.000605	2.70	0.18	2	20	721	19481
ALK6	rs34970181_A	BMPR1B	0.00143	3.24	0.0066	6	50	717	19447
ALK6	rs35973133_A	BMPR1B	0.000622	0	0.95	0	22	723	19479
ALK6	rs138801821_A	BMPR1B	0.000521	0	0.95	0	21	723	19480

Full uncut gels

Full unedited gel for Figure 4B DGAT2

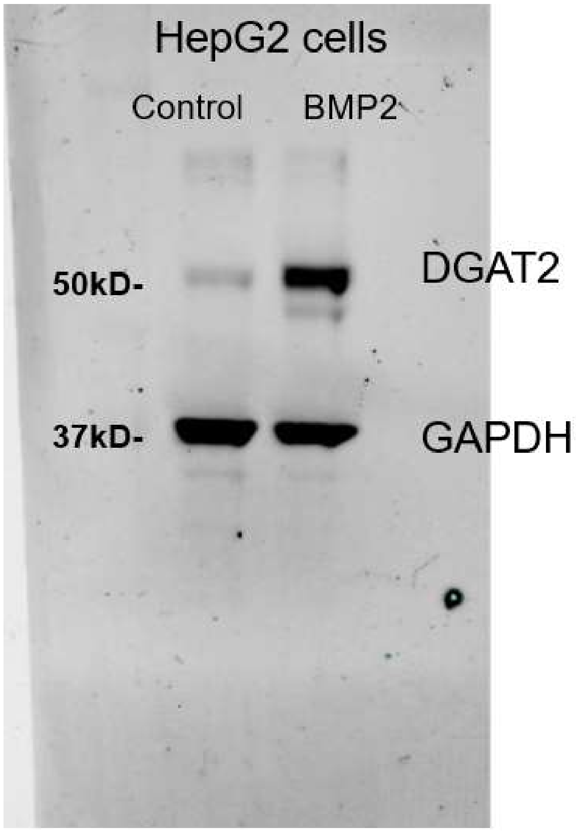

Full unedited gel for Figure 5A pSMAD 1/5

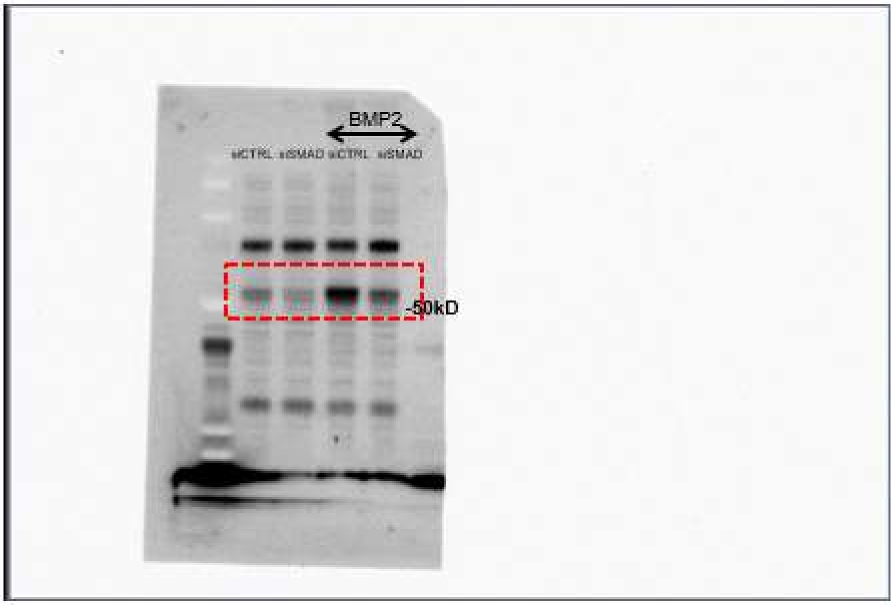

Full unedited gel for Figure 5A Total SMAD

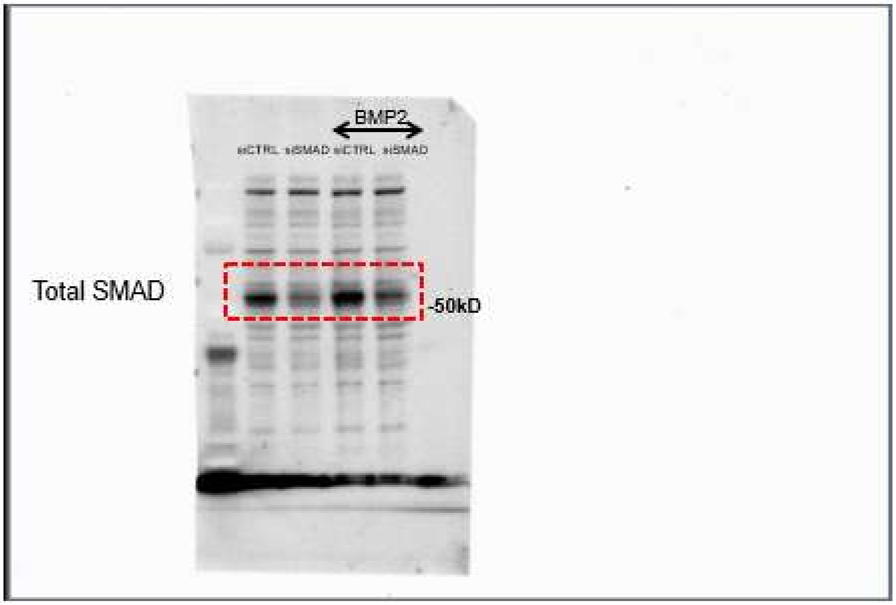

Full unedited gel for Figure 6d

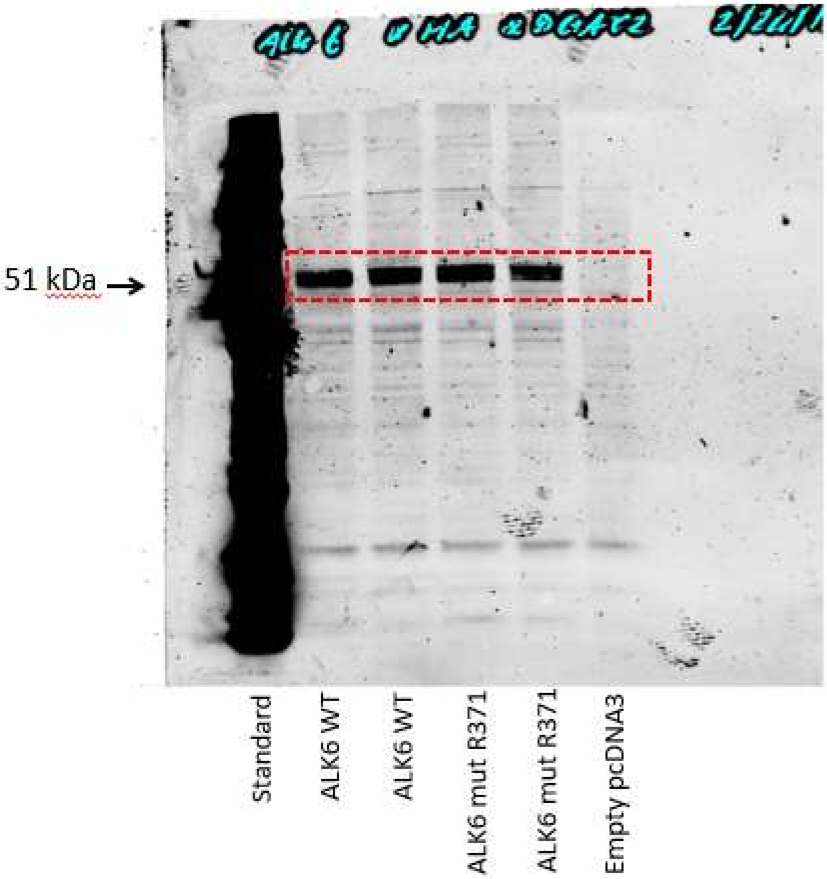

Full unedited gel for Figure 6d cont

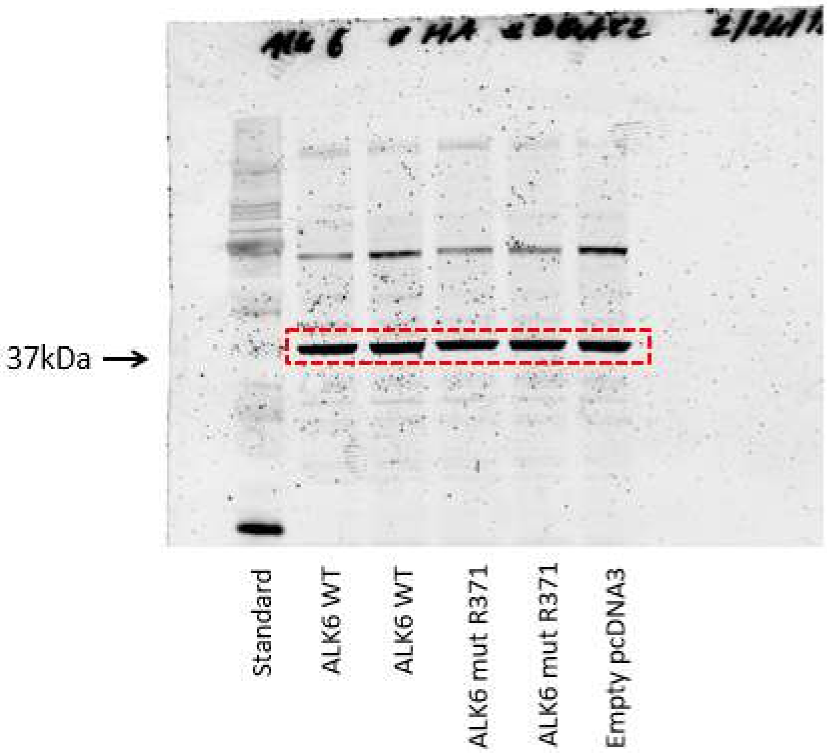

Full unedited gel for Figure 6e

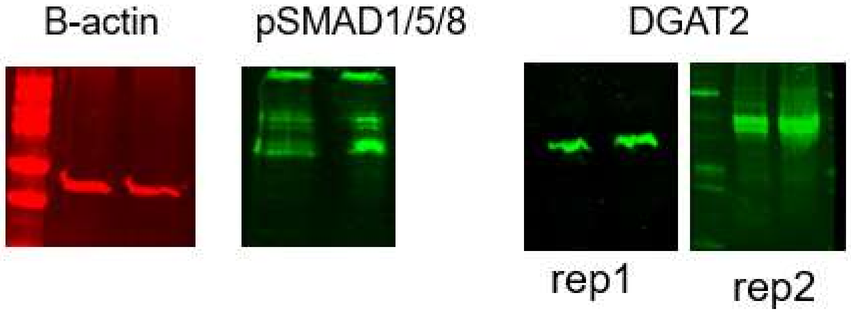

Full unedited gels for Supplemental Figure 7

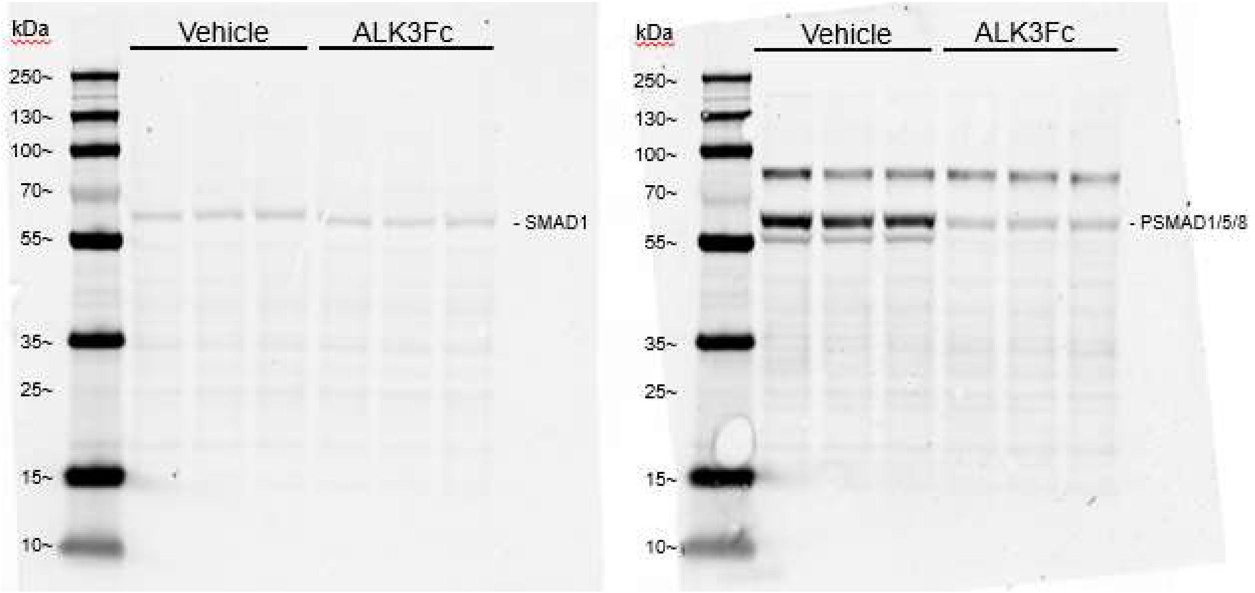

Sample Name	Target Name	Task	Reporter	CT (dilution corrected)		Sample Name	Target Name	Task	Reporter	CT	% ChIP vs Input	Sample Name
DMSO	dgat2	input	SYBR	19.70		DMSO	dgat2	Chip-ed	SYBR	32.68	**0.012**	DMSO
DMSO	dgat2	input	SYBR	20.17		DMSO	dgat2	Chip-ed	SYBR	30.29	**0.090**	DMSO
DMSO	dgat2	input	SYBR	19.12		DMSO	dgat2	Chip-ed	SYBR	32.25	**0.011**	DMSO
BMP	dgat2	input	SYBR	18.73		BMP	dgat2	Chip-ed	SYBR	27.56	**0.219**	BMP
BMP	dgat2	input	SYBR	21.38		BMP	dgat2	Chip-ed	SYBR	30.55	**0.174**	BMP
BMP	dgat2	input	SYBR	17.96		BMP	dgat2	Chip-ed	SYBR	26.63	**0.246**	BMP
LDN	dgat2	input	SYBR	17.31		LDN	dgat2	Chip-ed	SYBR	30.68	**0.010**	LDN
LDN	dgat2	input	SYBR	23.30		LDN	dgat2	Chip-ed	SYBR	33.21	**0.104**	LDN
LDN	dgat2	input	SYBR	20.63		LDN	dgat2	Chip-ed	SYBR	30.60	**0.100**	LDN
IgG	dgat2	input	SYBR	21.11		IgG	dgat2	Chip-ed	SYBR	38.82	**0.000**	IgG
IgG	dgat2	input	SYBR	20.66		IgG	dgat2	Chip-ed	SYBR	38.29	**0.000**	IgG
IgG	dgat2	input	SYBR	29.11		IgG	dgat2	Chip-ed	SYBR	>40	**0.000**	IgG

Absolute real time PCR values for CHIP results from Figure 5D

Sequencing data of expression constructs of ALK6R371Q (Sbjct) versus wild-type ALK6R (Query)

PREDICTED: Mus musculus bone morphogenetic protein receptor, type 1B (Bmpr1b), transcript variant X7, mRNA

Sequence ID: XM_006500943.3Length: 6070Number of Matches: 1

Related Information

Range 1: 1083 to 2588GenBankGraphicsNext MatchPrevious Match

Score	Expect	Identities	Gaps	Strand
2771 bits(1500)	0.0	1504/1506(99%)	0/1506(0%)	Plus/Plus

```
Query  1     ATGCTCTTACGAAGCTCTGGAAAATTAAATGTGGGCACCAAGAAGGAGGATGGAGAGAGT  60
             ||||||||||||||||||||||||||||||||||||||||||||||||||||||||||||
Sbjct  1083  ATGCTCTTACGAAGCTCTGGAAAATTAAATGTGGGCACCAAGAAGGAGGATGGAGAGAGT  1142

Query  61    ACAGCCCCCACCCCTCGGCCCAAGATCCTACGTTGTAAATGCCACCACCACTGTCCGGAA  120
             ||||||||||||||||||||||||||||||||||||||||||||||||||||||||||||
Sbjct  1143  ACAGCCCCCACCCCTCGGCCCAAGATCCTACGTTGTAAATGCCACCACCACTGTCCGGAA  1202

Query  121   GACTCAGTCAACAATATCTGCAGCACAGATGGGTACTGCTTCACGATGATAGAAGAAGAT  180
             ||||||||||||||||||||||||||||||||||||||||||||||||||||||||||||
Sbjct  1203  GACTCAGTCAACAATATCTGCAGCACAGATGGGTACTGCTTCACGATGATAGAAGAAGAT  1262

Query  181   GACTCTGGAATGCCTGTTGTCACCTCTGGATGTCTAGGACTAGAAGGGTCAGATTTTCAA  240
             ||||||||||||||||||||||||||||||||||||||||||||||||||||||||||||
Sbjct  1263  GACTCTGGAATGCCTGTTGTCACCTCTGGATGTCTAGGACTAGAAGGGTCAGATTTTCAA  1322

Query  241   TGTCGTGACACTCCCATTCCTCATCAAAGAAGATCAATTGAATGCTGCACAGAAAGGAAT  300
             ||||||||||||||||||||||||||||||||||||||||||||||||||||||||||||
Sbjct  1323  TGTCGTGACACTCCCATTCCTCATCAAAGAAGATCAATTGAATGCTGCACAGAAAGGAAT  1382

Query  301   GAGTGTAATAAAGACCTCCACCCCACTCTGCCTCCTCTCAAGGACAGAGATTTTGTTGAT  360
             ||||||||||||||||||||||||||||||||||||||||||||||||||||||||||||
Sbjct  1383  GAGTGTAATAAAGACCTCCACCCCACTCTGCCTCCTCTCAAGGACAGAGATTTTGTTGAT  1442

Query  361   GGGCCCATACACCACAAGGCCTTGCTTATCTCTGTGACTGTCTGTAGTTTACTCTTGGTC  420
             ||||||||||||||||||||||||||||||||||||||||||||||||||||||||||||
Sbjct  1443  GGGCCCATACACCACAAGGCCTTGCTTATCTCTGTGACTGTCTGTAGTTTACTCTTGGTC  1502
```

```
Query  421   CTCATTATTTTATTCTGTTACTTCAGGTATAAAAGACAAGAAGCCCGACCTCGGTACAGC  480
             ||||||||||||||||||||||||||||||||||||||||||||||||||||||||||||
Sbjct  1503  CTCATTATTTTATTCTGTTACTTCAGGTATAAAAGACAAGAAGCCCGACCTCGGTACAGC  1562

Query  481   ATTGGGCTGGAGCAGGACGAGACATACATTCCTCCTGGAGAGTCCCTGAGAGACTTGATC  540
             ||||||||||||||||||||||||||||||||||||||||||||||||||||||||||||
Sbjct  1563  ATTGGGCTGGAGCAGGACGAGACATACATTCCTCCTGGAGAGTCCCTGAGAGACTTGATC  1622

Query  541   GAGCAGTCTCAGAGCTCGGGAAGTGGATCAGGCCTCCCTCTGCTGGTCCAAAGGACAATA  600
             ||||||||||||||||||||||||||||||||||||||||||||||||||||||||||||
Sbjct  1623  GAGCAGTCTCAGAGCTCGGGAAGTGGATCAGGCCTCCCTCTGCTGGTCCAAAGGACAATA  1682

Query  601   GCTAAGCAAATTCAGATGGTGAAGCAGATTGGAAAAGGCCGCTATGGCGAGGTGTGGATG  660
             ||||||||||||||||||||||||||||||||||||||||||||||||||||||||||||
Sbjct  1683  GCTAAGCAAATTCAGATGGTGAAGCAGATTGGAAAAGGCCGCTATGGCGAGGTGTGGATG  1742

Query  661   GGAAAGTGGCGTGGAGAAAAGGTGGCTGTGAAAGTGTTCTTCACCACGGAGGAAGCCAGC  720
             ||||||||||||||||||||||||||||||||||||||||||||||||||||||||||||
Sbjct  1743  GGAAAGTGGCGTGGAGAAAAGGTGGCTGTGAAAGTGTTCTTCACCACGGAGGAAGCCAGC  1802

Query  721   TGGTTCCGAGAGACTGAGATATATCAGACGGTCCTGATGCGGCATGAGAATATTCTGGGG  780
             ||||||||||||||||||||||||||||||||||||||||||||||||||||||||||||
Sbjct  1803  TGGTTCCGAGAGACTGAGATATATCAGACGGTCCTGATGCGGCATGAGAATATTCTGGGG  1862

Query  781   TTCATTGCTGCAGATATCAAAGGGACTGGGTCCTGGACTCAGTTGTACCTCATCACAGAC  840
             ||||||||||||||||||||||||||||||||||||||||||||||||||||||||||||
Sbjct  1863  TTCATTGCTGCAGATATCAAAGGGACTGGGTCCTGGACTCAGTTGTACCTCATCACAGAC  1922

Query  841   TATCATGAAAACGGCTCCCTTTATGACTATCTGAAATCCACCACCTTAGACGCAAAGTCC  900
             ||||||||||||||||||||||||||||||||||||||||||||||||||||||||||||
Sbjct  1923  TATCATGAAAACGGCTCCCTTTATGACTATCTGAAATCCACCACCTTAGACGCAAAGTCC  1982

Query  901   ATGCTAAAGCTAGCCTACTCCTCTGTCAGCGGCCTATGCCATTTACACACGGAAATCTTT  960
             ||||| ||||||||||||||||||||||||||||||||||||||||||||||||||||||
Sbjct  1983  ATGCTGAAGCTAGCCTACTCCTCTGTCAGCGGCCTATGCCATTTACACACGGAAATCTTT  2042

Query  961   AGCACTCAAGGCAAGCCAGCAATCGCCCATCGAGACTTGAAAAGTAAAAACATCCTGGTG  1020
             ||||||||||||||||||||||||||||||||||||||||||||||||||||||||||||
Sbjct  2043  AGCACTCAAGGCAAGCCAGCAATCGCCCATCGAGACTTGAAAAGTAAAAACATCCTGGTG  2102
```

```
Query  1021  AAGAAAAAATGGAACTTGCTGCATAGCAGACCTGGGCTTGGCTGTCAAGTTCATTAGTGAC  1080
             ||||||||||||||||||||||||||||||||||||||||||||||||||||||||||||
Sbjct  2103  AAGAAAAAATGGAACTTGCTGCATAGCAGACCTGGGCTTGGCTGTCAAGTTCATTAGTGAC  2162

Query  1081  ACAAATGAGGTTGACATCCCACCCAACACCCAGGTTGGCACCAAGCGCTATATGCCTCCA  1140
             ||||||||||||||||||||||||||||||| ||||||||||||||||||||||||||||
Sbjct  2163  ACAAATGAGGTTGACATCCCACCCAACACCCGGGTTGGCACCAAGCGCTATATGCCTCCA  2222

Query  1141  GAAGTGCTGGACGAGAGCTTGAATAGAAACCATTTCCAGTCCTACATTATGGCTGACATG  1200
             ||||||||||||||||||||||||||||||||||||||||||||||||||||||||||||
Sbjct  2223  GAAGTGCTGGACGAGAGCTTGAATAGAAACCATTTCCAGTCCTACATTATGGCTGACATG  2282

Query  1201  TACAGCTTTGGACTCATCCTCTGGGAGATTGCAAGGAGATGTGTTTCTGGAGGTATAGTG  1260
             ||||||||||||||||||||||||||||||||||||||||||||||||||||||||||||
Sbjct  2283  TACAGCTTTGGACTCATCCTCTGGGAGATTGCAAGGAGATGTGTTTCTGGAGGTATAGTG  2342

Query  1261  GAAGAATACCAGCTTCCCTATCACGACCTGGTGCCCAGTGACCCTTCTTATGAGGACATG  1320
             ||||||||||||||||||||||||||||||||||||||||||||||||||||||||||||
Sbjct  2343  GAAGAATACCAGCTTCCCTATCACGACCTGGTGCCCAGTGACCCTTCTTATGAGGACATG  2402

Query  1321  AGAGAAATTGTGTGCATGAAGAAGTTACGGCCTTCATTCCCCAATCGATGGAGCAGTGAT  1380
             ||||||||||||||||||||||||||||||||||||||||||||||||||||||||||||
Sbjct  2403  AGAGAAATTGTGTGCATGAAGAAGTTACGGCCTTCATTCCCCAATCGATGGAGCAGTGAT  2462

Query  1381  GAGTGTCTCAGGCAGATGGGGAAGCTTATGACAGAGTGCTGGGCGCAGAATCCTGCCTCC  1440
             ||||||||||||||||||||||||||||||||||||||||||||||||||||||||||||
Sbjct  2463  GAGTGTCTCAGGCAGATGGGGAAGCTTATGACAGAGTGCTGGGCGCAGAATCCTGCCTCC  2522

Query  1441  AGGCTGACGGCCCTGAGAGTTAAGAAAACCCTTGCCAAAATGTCAGAGTCCCAGGACATT  1500
             ||||||||||||||||||||||||||||||||||||||||||||||||||||||||||||
Sbjct  2523  AGGCTGACGGCCCTGAGAGTTAAGAAAACCCTTGCCAAAATGTCAGAGTCCCAGGACATT  2582

Query  1501  AAACTC  1506
             ||||||
Sbjct  2583  AAACTC  2588
```

bone morphogenetic protein receptor type-1B isoform a precursor [Mus musculus]

Sequence ID: NP_031586.1Length: 502Number of Matches: 1

See 19 more title(s)

Related Information

Gene-associated gene details

Map Viewer-aligned genomic context

Identical Proteins-Identical proteins to NP_031586.1

Range 1: 1 to 502GenPeptGraphicsNext MatchPrevious Match

Score	Expect	Method	Identities	Positives	Gaps
1045 bits(2703)	0.0	Compositional matrix adjust.	501/502(99%)	502/502(100%)	0/502(0%)

```
Query  1    MLLRSSGKLNVGTKKEDGESTAPTPRPKILRCKCHHHCPEDSVNNICSTDGYCFTMIEED  60
            MLLRSSGKLNVGTKKEDGESTAPTPRPKILRCKCHHHCPEDSVNNICSTDGYCFTMIEED
Sbjct  1    MLLRSSGKLNVGTKKEDGESTAPTPRPKILRCKCHHHCPEDSVNNICSTDGYCFTMIEED  60

Query  61   DSGMPVVTSGCLGLEGSDFQCRDTPIPHQRRSIECCTERNECNKDLHPTLPPLKDRDFVD  120
            DSGMPVVTSGCLGLEGSDFQCRDTPIPHQRRSIECCTERNECNKDLHPTLPPLKDRDFVD
Sbjct  61   DSGMPVVTSGCLGLEGSDFQCRDTPIPHQRRSIECCTERNECNKDLHPTLPPLKDRDFVD  120

Query  121  GPIHHKALLISVTVCSLLLVLIILFCYFRYKRQEARPRYSIGLEQDETYIPPGESLRDLI  180
            GPIHHKALLISVTVCSLLLVLIILFCYFRYKRQEARPRYSIGLEQDETYIPPGESLRDLI
Sbjct  121  GPIHHKALLISVTVCSLLLVLIILFCYFRYKRQEARPRYSIGLEQDETYIPPGESLRDLI  180

Query  181  EQSQSSGSGSGLPLLVQRTIAKQIQMVKQIGKGRYGEVWMGKWRGEKVAVKVFFTTEEAS  240
            EQSQSSGSGSGLPLLVQRTIAKQIQMVKQIGKGRYGEVWMGKWRGEKVAVKVFFTTEEAS
Sbjct  181  EQSQSSGSGSGLPLLVQRTIAKQIQMVKQIGKGRYGEVWMGKWRGEKVAVKVFFTTEEAS  240

Query  241  WFRETEIYQTVLMRHENILGFIAADIKGTGSWTQLYLITDYHENGSLYDYLKSTTLDAKS  300
            WFRETEIYQTVLMRHENILGFIAADIKGTGSWTQLYLITDYHENGSLYDYLKSTTLDAKS
Sbjct  241  WFRETEIYQTVLMRHENILGFIAADIKGTGSWTQLYLITDYHENGSLYDYLKSTTLDAKS  300

Query  301  MLKLAYSSVSGLCHLHTEIFSTQGKPAIAHRDLKSKNILVKKNGTCCIADLGLAVKFISD  360
            MLKLAYSSVSGLCHLHTEIFSTQGKPAIAHRDLKSKNILVKKNGTCCIADLGLAVKFISD
Sbjct  301  MLKLAYSSVSGLCHLHTEIFSTQGKPAIAHRDLKSKNILVKKNGTCCIADLGLAVKFISD  360

Query  361  TNEVDIPPNTQVGTKRYMPPEVLDESLNRNHFQSYIMADMYSFGLILWEIARRCVSGGIV  420
            TNEVDIPPNT+VGTKRYMPPEVLDESLNRNHFQSYIMADMYSFGLILWEIARRCVSGGIV
Sbjct  361  TNEVDIPPNTRVGTKRYMPPEVLDESLNRNHFQSYIMADMYSFGLILWEIARRCVSGGIV  420
```

```
Query  421  EEYQLPYHDLVPSDPSYEDMREIVCMKKLRPSFPNRWSSDECLRQMGKLMTECWAQNPAS  480
            EEYQLPYHDLVPSDPSYEDMREIVCMKKLRPSFPNRWSSDECLRQMGKLMTECWAQNPAS
Sbjct  421  EEYQLPYHDLVPSDPSYEDMREIVCMKKLRPSFPNRWSSDECLRQMGKLMTECWAQNPAS  480

Query  481  RLTALRVKKTLAKMSESQDIKL  502
            RLTALRVKKTLAKMSESQDIKL
Sbjct  481  RLTALRVKKTLAKMSESQDIKL  502
```

Supplemental References

1 Cuny, G. D. *et al.* Structure-activity relationship study of bone morphogenetic protein (BMP) signaling inhibitors. *Bioorg Med Chem Lett* **18**, 4388-4392, doi:10.1016/j.bmcl.2008.06.052 (2008).

2 Chomczynski, P. & Sacchi, N. Single-step method of RNA isolation by acid guanidinium thiocyanate-phenol-chloroform extraction. *Anal. Biochem.* **162**, 156-159, doi:10.1006/abio.1987.99990003-2697(87)90021-2 [pii] (1987).

3 Kim, T. H. & Dekker, J. ChIP-Quantitative Polymerase Chain Reaction (ChIP-qPCR). *Cold Spring Harbor protocols* **2018**, pdb.prot082628, doi:10.1101/pdb.prot082628 (2018).

4 McFie, P. J. & Stone, S. J. A fluorescent assay to quantitatively measure in vitro acyl CoA:diacylglycerol acyltransferase activity. *J Lipid Res* **52**, 1760-1764, doi:10.1194/jlr.D016626 [pii] (2011).

5 Nishihara, A., Watabe, T., Imamura, T. & Miyazono, K. Functional heterogeneity of bone morphogenetic protein receptor-II mutants found in patients with primary pulmonary hypertension. *Molecular biology of the cell* **13**, 3055-3063, doi:10.1091/mbc.E02-02-0063 (2002).

6 Roden, D. M. *et al.* Development of a large-scale de-identified DNA biobank to enable personalized medicine. *Clinical pharmacology and therapeutics* **84**, 362-369, doi:10.1038/clpt.2008.89 (2008).

7 Denny, J. C. *et al.* PheWAS: demonstrating the feasibility of a phenome-wide scan to discover gene-disease associations. *Bioinformatics (Oxford, England)* **26**, 1205-1210, doi:10.1093/bioinformatics/btq126 (2010).

8 Denny, J. C. *et al.* Systematic comparison of phenome-wide association study of electronic medical record data and genome-wide association study data. *Nat Biotechnol* **31**, 1102-1110, doi:10.1038/nbt.2749 (2013).

9 Weston, S. R. *et al.* Racial and ethnic distribution of nonalcoholic fatty liver in persons with newly diagnosed chronic liver disease. *Hepatology (Baltimore, Md.)* **41**, 372-379, doi:10.1002/hep.20554 (2005).

10 Pan, J. J. & Fallon, M. B. Gender and racial differences in nonalcoholic fatty liver disease. *World journal of hepatology* **6**, 274-283, doi:10.4254/wjh.v6.i5.274 (2014).
